# Electrochemical generation of 2,3-oxazolidinone glycosyl triflates as an intermediate for stereoselective glycosylation

**DOI:** 10.3762/bjoc.8.52

**Published:** 2012-03-28

**Authors:** Toshiki Nokami, Akito Shibuya, Yoshihiro Saigusa, Shino Manabe, Yukishige Ito, Jun-ichi Yoshida

**Affiliations:** 1 Department of Synthetic Chemistry and Biological Chemistry, Kyoto University, Nishikyo-ku Kyoto 615-8510 Japan; 2 Advanced Science Institute, RIKEN, Hirosawa, Wako, Saitama 351-0198, Japan; 3 ERATO JST, Hirosawa, Wako, Saitama 351-0198, Japan

**Keywords:** amino sugar, anomerization, electrochemical oxidation, glycosylation, thioglycoside

## Abstract

Glycosyl triflates with a 2,3-oxazolidinone protecting group were generated from thioglycosides by low-temperature electrochemical oxidation. The glycosyl triflates reacted with alcohols to give the corresponding glycosides β-selectively at low temperatures. However, α-selectivity was observed in the absence of base at elevated reaction temperatures. In situ generated triflic acid promotes the isomerization of β-products to α-products.

## Introduction

Stereoselective formation of glycosidic linkages is the key issue in oligosaccharide synthesis, because both 1,2-*trans* and 1,2-*cis* aminoglycosides are ubiquitous in biologically active oligosaccharides [1–5]. The 1,2-*trans* aminoglycosides, which are found in Nod factor [1] and lipid A [2], can be easily prepared by protecting the 2-amino group with phthaloyl or carbamate groups [6]. On the other hand, 1,2-*cis* glycosidic linkages are still difficult to make with perfect stereoselectivity. Although 2-azido-substituted glycosyl donors are commonly used for the preparation of 1,2-*cis* glycosidic linkages of amino sugars [7–8], the selectivity highly depends on the nature of the glycosyl acceptors and reaction conditions. In the last decade, 2,3-oxazolidinone protected 2-amino-2-deoxy-glycosides have been developed as glycosyl donors for the stereoselective synthesis of amino sugars [9–24]. These glycosyl donors afford the corresponding glycosides in a 1,2-*trans* or 1,2-*cis* selective manner by action with various types of glycosyl acceptors. However, it is still uncertain whether glycosyl triflate intermediates [25], which were detected by NMR, play an important role in the stereoselective formation of both α- and β-isomers.

We have developed an electrochemical method to generate and accumulate highly reactive glycosyl triflates by low-temperature electrochemical oxidation of thioglycosides [26–30]. Although Kerns and Ye have already reported a chemically generated glycosyl triflate equipped with an acetylated 2,3-oxazolidinone protecting group [11,19], we envisioned that the electrochemically generated glycosyl triflate with a 2,3-oxazolidinone protecting group could be a useful intermediate to reveal stereoselectivity in glycosylations via glycosyl triflate intermediates. In this paper, we report the generation, accumulation, and characterization by low-temperature NMR analyses, of the corresponding glycosyl triflates. Electrochemical glycosylation of the thioglycoside donor with 2,3-oxazolidinone protecting group gave both 1,2-*trans* linkages in the presence of a base at low temperatures and 1,2-*cis* glycosidic linkages in the absence of a base at elevated temperatures.

## Results and Discussion

**Scheme 1:**
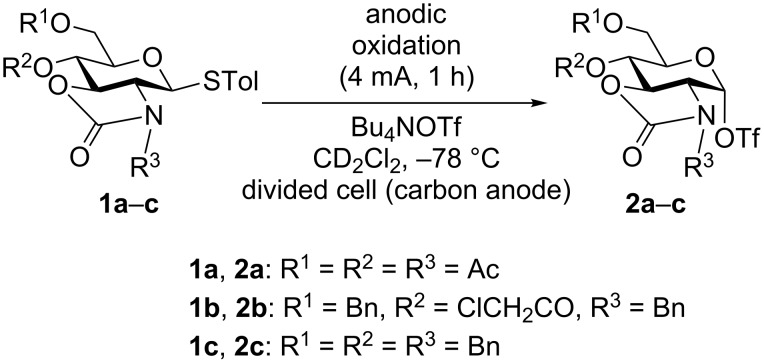
Electrochemical conversion of thioglycosides to glycosyl triflates.

**Figure 1:**
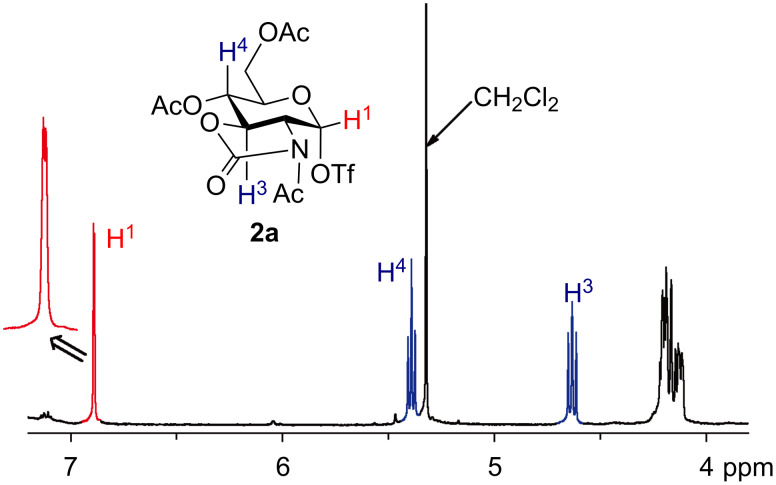
^1^H NMR spectrum of glycosyl triflate **2a**.

We began by conducting the electrochemical oxidation of 2*,*3-oxazolidinone thioglycosides **1a**–**1c** in the absence of a glycosyl acceptor at low temperatures in order to generate and accumulate the corresponding glycosyl triflates (Scheme 1). Low-temperature NMR measurements of the anodic solution were carried out to confirm the structure of the glycosyl triflates. For example, the anodic solution obtained by the electrochemical oxidation of thioglycoside **1a** (4 mA, 1 h) exhibited a single set of peaks for glycosyl triflate **2a** in the ^1^H NMR spectrum at −80 °C (Figure 1). In contrast to the previous reports by Kerns and Ye, the corresponding β-triflate was not observed under these conditions [11,19]. The small coupling constant of the anomeric proton (*J* = 2.1 Hz) indicates α-configuration of the anomeric triflate. The ^1^H and ^13^C NMR chemical shifts of the anomeric protons and carbons of glycosyl triflates **2a**–**c** are listed in Table 1. In all cases, the starting thioglycosides **1a**–**c** were quantitatively converted to the corresponding glycosyl triflates **2a**–**c,** which have α-configuration of the anomeric triflate. Although the chemical shift of the anomeric proton H-1 of glycosyl triflate **2a** appears at a lower chemical shift (6.89 ppm) than those of glycosyl triflates **2b** (5.97 ppm) and **2c** (5.95 ppm), the chemical shift of the anomeric carbon is around 100 ppm in all cases and the corresponding cross peaks of the anomeric protons and carbons were observed in HMQC spectra.

**Table 1:** ^1^H- and ^13^C NMR chemical shifts of the anomeric proton and carbon of glycosyl triflates **2a**–**c**.

entry	glycosyl triflate	^1^H NMR [δ (ppm)^a^, *J* (Hz)^b^]	^13^C NMR [δ (ppm)^a^]

1	**2a**	6.89, 2.1^c^	99.9
2	**2b**	5.97, singlet	99.9
3	**2c**	5.95, singlet	100.7

^a^chemical shift; ^b^coupling constant; ^c^doublet.

Using electrochemically generated glycosyl triflate **2a**, the stereoselectivity of the glycosylation was investigated by the addition of five equiv of various alcohols (Table 2). In accordance with our previous results [26,29], high β-selectvity was observed with highly reactive alcohols (Table 2, entries 1 and 2). On the other hand, less nucleophilic alcohols, such as benzyl alcohol and CF_3_CH_2_OH, gave the corresponding glycosides in lower β-selectivity than methanol or ethanol (Table 2, entries 3 and 4) [31].

**Table 2:** Glycosylation of electrochemically generated glycosyl triflate **2a**.

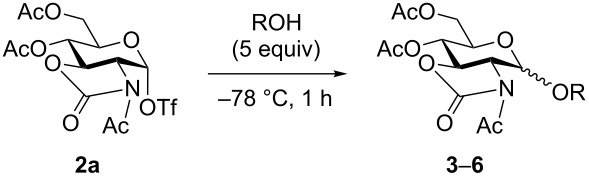		

entry	ROH	product: yield^a^, ratio (α to β)^b^

1	MeOH	**3**: 76% (<1 to >99)
2	EtOH	**4**: 71% (<1 to >99)
3	BnOH	**5**: 89% (9 to 91)
4	CF_3_CH_2_OH	**6**: 82% (15 to 85)

^a^isolated yields; ^b^determined by ^1^H NMR.

Next, we examined the electrochemical activation of thioglycoside **1a** to generate glycosyl triflate **2a** in the presence of glycosyl acceptor **7**. In order to improve the yield and the stereoselectivity of glycosylation, we examined the effects of the reaction temperature and base (Table 3). Although poor β-selectivity and yield were observed at low temperatures in the absence of a base (Table 3, entry 1), the addition of an organic base such as 2,6-di-*tert*-butyl-4-methylpyridine (DTBMP), significantly improved the β-selectivity and yield (Table 3, entry 2). In both cases small amounts of the α-isomers of the starting thioglycoside **9** and glucal **10** were obtained as byproducts (Table 3, entries 1 and 2). The yield was further increased and the formation of byproducts was suppressed by raising the reaction temperature to 0 °C (Table 3, entry 3). On the other hand, the corresponding α-isomer of disaccharide **8α** was obtained as a major product together with the anomerized donor **9** when the reaction was performed at 0 °C in the absence of DTBMP (Table 3, entry 4). These anomerizations may be caused by the endocyclic cleavage reaction [22–24], which is often observed for pyranosides with a 2,3-*trans* carbamate group, under acidic conditions. The yield of disaccharide **8** was improved by raising the temperature to 0 °C after the completion of electrolysis at −78 °C (Table 3, entry 5). The fact that disaccharide **8** was obtained in higher yields (59% to 78%) and that only a trace amount of α-isomer **9** (4%) was obtained strongly suggests that the isomerization of thioglycoside donor **1a** occurs during the electrolysis at 0 °C. It is noteworthy that the α- and β-glycosides could be selectively prepared from the same glycosyl donor with a 2,3-*trans* carbamate group, simply by changing the reaction conditions.

**Table 3:** Electrochemical glycosylation in the presence of glycosyl acceptor.

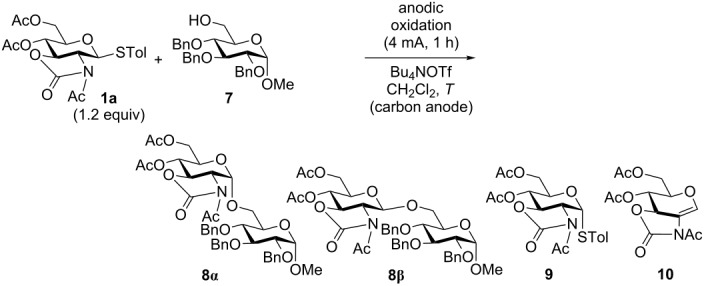					

entry	*T*	base	product: yield, ratio (**8α** to **8β**)^a^		

1	−78 °C	–	**8**^b^: 36% (46 to 54)	**9**: <3%^c^	**10**: 34%^c^
2	−78 °C	DTBMP^d^	**8**: 60% (4 to 96)	**9**: <3%^c^	**10**: 24%^c^
3	0 °C	DTBMP^d^	**8**: 79% (3 to 97)	**9**: trace	**10**: trace
4	0 °C	–	**8**: 59% (α only)	**9**: 29%^c^	**10**: 2%^c^
5	−78 °C then 0 °C^e^	–	**8**: 78% (α only)^f^	**9**: 4%^c^	**10**: 8%^c^

^a^determined by ^1^H NMR; ^b^**1a** (ca 15%) was recovered; ^c^yields are based on **1a**; ^d^5.0 equiv; ^e^the reaction temperature was raised after electrolysis; ^f^**1a** (ca 6%) was recovered.

**Scheme 2:**
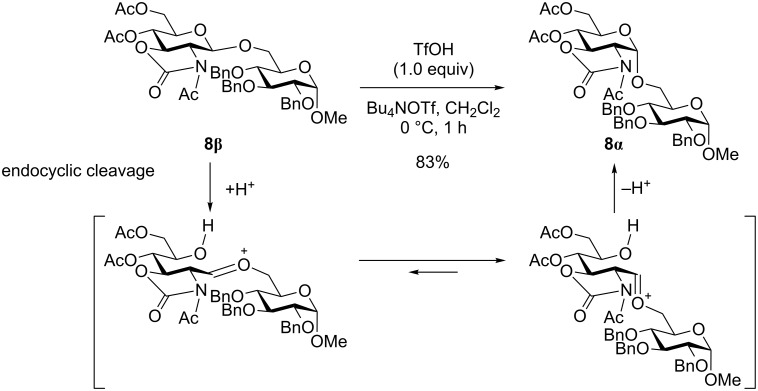
Triflic acid mediated isomerization of β-glycoside.

In order to confirm that the anomerization of **8β** to **8α** could take place under the reaction conditions, we examined the acid-mediated isomerization of the β-isomer of disaccharide **8β** to the α-isomer **8α** (Scheme 2). Triflic acid (TfOH) must be generated in situ by the reaction of glycosyl triflate **2a** with alcohols. Thus, TfOH (1.0 equiv) and tetrabutylammonium triflate (Bu_4_NOTf) (5.0 equiv), which was used as a supporting electrolyte for electrolysis, were added to a CH_2_Cl_2_ solution of the β-isomer of disaccharide **8β** at 0 °C. After stirring at 0 °C for 1 h, the reaction was quenched by the addition of Et_3_N. The α-isomer of the disaccharide **8α** was obtained in 83% yield as a single isomer. This experiment shows that the α-product **8α** is the isomerization product of the β-isomer **8β** as a result of endocyclic cleavage, as shown in Scheme 2.

## Conclusion

In conclusion, we have achieved the electrochemical generation and accumulation of glycosyl triflates equipped with the 2,3-oxazolidinone protecting group. This glycosyl triflate intermediate reacted with alcohols of high reactivity to afford β-glycosides as kinetic products. The α-products were also obtained at elevated temperatures after anomerization of the β-products, as both Oscarson and our group have previously reported [18,22–24]. Namely, α- and β-glycosides were obtained from 2,3-oxazolidinone donors by changing the reaction conditions. Further investigations to reveal the scope and limitations of the glycosylation reaction using electrochemically generated glycosyl triflates are in progress in our laboratory.

## Supporting Information

File 1 Experimental procedures, spectral data of glycosyl triflates and new compounds, and ^1^H- and ^13^C NMR spectra.
