# Supplementary material for: Electrochemical generation of 2,3-oxazolidinone glycosyl triflates as an intermediate for stereoselective glycosylation
Source: Beilstein J Org Chem. 2012 Mar 28;8:456–60. doi: 10.3762/bjoc.8.52 (PMC3326625; doi:10.3762/bjoc.8.52)
Supplement: File 1 — Experimental procedures, spectral data of glycosyl triflates and new compounds, and 1H- and 13C NMR spectra. [file Beilstein_J_Org_Chem-08-456-s001.pdf]

Supporting Information

for

Electrochemical generation of 2,3-oxazolidinone glycosyl triflates as an intermediate for stereoselective glycosylation

Toshiki Nokami[1], Akito Shibuya[1], Yoshihiro Saigusa[1], Shino Manabe*[2], Yukishige Ito[2,3] and Jun-ichi Yoshida*[1]

Address: [1]Department of Synthetic Chemistry and Biological Chemistry, Kyoto University, Nishikyo-ku Kyoto 615-8510 Japan; [2]Advanced Science Institute, RIKEN, Hirosawa, Wako, Saitama 351-0198, Japan and [3]ERATO JST, Hirosawa, Wako, Saitama 351-0198, Japan

Email: Jun-ichi Yoshida* - yoshida@sbchem.kyoto-u.ac.jp and Shino Manabe* - smanabe@riken.jp

* Corresponding author

Experimental procedures, spectral data of glycosyl triflates and new compounds, and ^{1}H- and ^{13}C NMR spectra

Contents

1.	General	S2
2.	Preparation of glycosyl donor	S2–S3
3.	Procedures for low-temperature NMR analysis of the glycosyl triflate	S3–S4
4.	Glycosylation of glycosyl triflate with alcohols	S4–S6
5.	Electrochemical glycosylation in the presence of glycosyl acceptor	S6–S8
6.	Triflic acid mediated isomerization of β-isomer to α-isomer	S8
7.	References	S8
8.	^{1}H and ^{13}C NMR spectra of thioglycoside **1b**	S9–S10
9.	^{1}H, ^{13}C NMR, ^{1}H/^{1}H COSY, and HMQC spectra of glycosyl triflate **2a**	S11–S14
10.	^{1}H, ^{13}C NMR, ^{1}H/^{1}H COSY, and HMQC spectra of glycosyl triflate **2b**	S15–S18
11.	^{1}H, ^{13}C NMR, ^{1}H/^{1}H COSY, and HMQC spectra of glycosyl triflate **2c**	S19–S22
12.	^{1}H and ^{13}C NMR spectra of β-methyl glycoside **3β**	S23–S24
13.	^{1}H and ^{13}C NMR spectra of β-ethyl glycoside **4β**	S25–S26
14.	^{1}H and ^{13}C NMR spectra of α-benzyl glycoside **5α**	S27–S28
15.	^{1}H and ^{13}C NMR spectra of α/β-trifluoroethyl glycoside **6α/6β**	S29–S32
16.	^{1}H and ^{13}C NMR spectra of α-thioglycoside **9**	S33–S34
17.	^{1}H and ^{13}C NMR spectra of α-thioglycoside **10**	S35–S36

1. General

^{1}H and ^{13}C NMR spectra were recorded on Varian MERCURYplus-400 (^{1}H, 400 MHz, ^{13}C, 100 MHz). Low-temperature ^{1}H, ^{13}C NMR, and ^{13}C/^{1}H HMQC spectra were recorded on JEOL ECA-600P (^{1}H, 600 MHz, ^{13}C, 150 MHz). EI and CI mass spectra were recorded on JEOL JMS-SX102A mass spectrometers. FAB and ESI mass spectra were recorded on JEOL JMS-HX110A and Thermo EXACTIVE mass spectrometers, respectively. Unless otherwise noted, all materials were obtained from commercial suppliers and used without further purification. Dichloromethane was washed with water, distilled from P_2O_5, redistilled from dried K_2CO_3 to remove a trace amount of acid, and stored over molecular sieves (4 Å). Bu_4NOTf was dried over P_2O_5 under vacuum and CD_2Cl_2 was dried over molecular sieves (4 Å) before use. Starting material **S1** [1], glycosyl donor **1a** [2], **1c** [1], and glycosyl acceptors **7** [3] were prepared according to the reported procedures.

2. Preparation of glycosyl donor

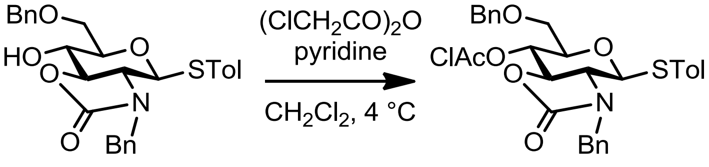

S1 **1b**

To a solution of alcohol **S1** (300 mg, 0.611 mol) in pyridine (97 μL, 1.22 mmol) and CH_2Cl_2 (1 mL), chloroacetic anhydride (92 mg, 0.733 mmol) was added at 4 °C. After 3 h, 0.5 M HCl aq. was added. The aqueous layer was extracted with EtOAc. The combined layers were washed with brine. After the extract was dried over Na_2SO_4, the solvent was removed in vacuo. The residue was purified by silica gel column chromatography (hexane:EtOAc 4:1 to 7:3) to give the chloroacetate **1b** (340 mg, 98%) as a colorless oil. ***p*-Tolyl *N*-benzyl-2-amino-6-*O*-benzyl-2,3-*N*,*O*-carbonyl-4-*O*-chloroacetyl-2-deoxy-1-thio-β-D-glucopyranoside (1b).** ^{1}H NMR ($CDCl_3$, 400 MHz) δ 7.38–7.22 (m, 12H), 7.01 (d, *J* = 7.6 Hz, 2H), 5.33 (dd, *J* = 10.4, 8.4 Hz, 1H), 4.72 (s, 2H), 4.71 (d, *J* = 8.4 Hz, 1H), 4.52 (d, *J* = 11.6 Hz, 1H), 4.44 (d, *J* = 12.0 Hz, 1H), 4.15 (*pseudo* t, *J* = 10.8 Hz, 1H), 3.97 (d, *J* = 14.8 Hz, 1H), 3.89 (d, *J* = 15.2 Hz, 1H), 3.68 (dd, *J* = 8.4, 3.2 Hz, 1H), 3.63 (dd, *J* = 10.8, 3.2 Hz, 1H), 3.58 (dd, *J* = 10.8, 4.8 Hz, 1H), 3.52 (dd, *J* = 11.2, 9.2 Hz, 1H), 2.30 (s, 3H). ^{13}C NMR ($CDCl_3$, 100 MHz) δ 165.8, 158.5, 150.3, 138.9, 137.4, 135.9, 133.1, 129.9, 128.7, 128.4, 128.2, 128.1, 127.94, 127.85, 127.7, 87.0, 79.5, 78.2, 73.6, 69.6, 68.5, 60.1, 47.5, 40.4, 21.1. HRMS (ESI) *m/z* calcd for $C_{30}H_{31}ClNO_6S$ $[M+H]^+$, 568.1555; found, 568.1560.

3. Low-temperature NMR analysis of glycosyl triflates

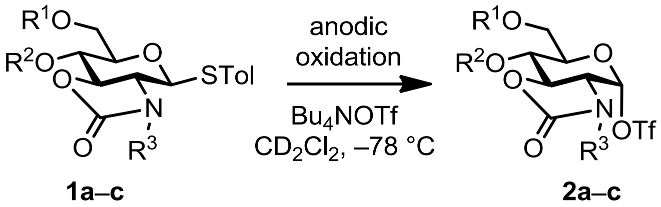

1a, **2a**: R^1 = R^2 = R^3 = Ac
1b, **2b**: R^1 = Bn, R^2 = $ClCH_2CO$, R^3 = Bn
1c, **2c**: R^1 = R^2 = R^3 = Bn

The anodic oxidation was carried out in an H-type divided cell (4G glass filter) equipped with a carbon felt anode (Nippon Carbon JF-20-P7, ca. 160 mg, dried at 250 °C/1 mmHg before use) and a platinum plate cathode (10 mm x 10 mm). In the anodic chamber were placed thioglycoside **1a** (45.0 mg, 0.10 mmol) and 0.1 M Bu_4NOTf in CD_2Cl_2 (5.0 mL). In the cathodic chamber were placed trifluoromethanesulfonic acid (22 μL, 0.25 mmol) and 0.1 M Bu_4NOTf in CD_2Cl_2 (5.0 mL). The constant current electrolysis (4.0 mA) was carried out at −78 °C with magnetic stirring. After 1.5 F/mol of electricity was consumed, the reaction mixture of the anodic chamber was transferred to a 5 mm NMR tube with a septum cap under an argon atmosphere at −78 °C. The NMR measurement was carried out at −80 °C. Chemical shifts were reported using signals of CH_2Cl_2 at 5.32 ppm (^{1}H NMR) and CD_2Cl_2 at 53.8 ppm (^{13}C NMR) as standards. **Triflyl 2-*N*-acetyl-4,6-di-*O*-acetyl-2,3-*N*,*O*-carbonyl-2-deoxy-α-D-glucopyranoside** (**2a**). Selected data for **2a** (7.0–2.0 ppm for ^{1}H NMR and 100–20 ppm for ^{13}C NMR). ^{1}H NMR (CD_2Cl_2, 600 MHz) δ 6.89 (d, *J* = 2.1 Hz, 1H, H-1), 5.39 (dd, *J* = 10.3, 9.6 Hz, 1H, H-4), 4.63 (dd, *J* = 12.4, 10.3 Hz, 1H, H-3), 4.21–4.16 (m, 3H), 4.14–4.11 (m, 1H, H-5), 2.45 (s, 3H), 2.10 (s, 3H), 2.04 (s, 3H). ^{13}C NMR (CD_2Cl_2, 150 MHz) δ 99.9 (C-1), 72.9 (C-5), 72.7 (C-3), 65.7 (C-4), 60.1 (C-6), 57.6 (C-2), 23.6 (CH_3 of NAc), 20.60 (CH_3 of OAc), 20.52 (CH_3 of OAc).

The anodic oxidation of thioglycoside **1b** (54.5 mg, 0.0984 mmol) afforded **triflyl 2-*N*-benzyl-6-*O*-benzyl-2,3-*N*,*O*-carbonyl-4-*O*-chloroacetyl-2-deoxy-α-D-glucopyranoside** (**2b**). Selected data for **2b** (6.0–3.5 ppm for ^{1}H NMR and 100–40 ppm for ^{13}C NMR). ^{1}H NMR (CD_2Cl_2, 600 MHz) δ 5.97 (s, 1H, H-1), 5.51 (dd, *J* = 10.3, 9.6 Hz, 1H, H-4), 4.62 (d, *J* = 15.1 Hz, 1H, CH_2Ph), 4.52 (d, *J* = 11.7 Hz, 1H, C(O)CH_2Cl), 4.51 (d, *J* = 11.7 Hz, 1H, H-3), 4.23 (d, *J* = 12.4 Hz, 1H, C(O)CH_2Cl), 4.19 (d, *J* = 14.4 Hz, 1H, CH_2Ph), 4.06 (d, *J* = 16.5 Hz, 1H, CH_2Ph), 3.91 (d, *J* = 8.9 Hz, 1H, H-5), 3.79 (d, *J* = 16.5 Hz, 1H, CH_2Ph), 3.58 (d, *J* = 12.4 Hz, 1H, H-2), 3.51 (d, *J* = 11.7 Hz, 1H, H-6), 3.40 (d, *J* = 11.0 Hz, 1H, H-6'). ^{13}C NMR (CD_2Cl_2, 150 MHz) δ 99.9 (C-1), 74.3 (C-5), 72.2 (C-3, C(O)*C*H_2Cl), 67.3 (C-4), 64.0 (C-6), 58.4 (C-2), 47.8 (*C*H_2Ph), 40.9 (*C*H_2Ph)

The anodic oxidation of thioglycoside **1c** (57.9 mg, 0.0995 mmol) afforded **triflyl 2-*N*-benzyl-4,6-di-*O*-benzyl-2,3-*N*,*O*-carbonyl-2-deoxy-α-D-glucopyranoside** (**2c**). Selected data for **1c** (6.0–3.4 ppm for ^{1}H NMR and 110–40 ppm for ^{13}C NMR). ^{1}H NMR (CD_2Cl_2, 600 MHz) δ 5.95 (s, 1H, H-1), 4.78 (d, *J* = 11.0 Hz, 1H, CH_2Ph), 4.59–4.55 (m, 2H, H-3, CH_2Ph), 4.42 (d, *J* = 11.0 Hz, 1H, CH_2Ph), 4.40 (d, *J* = 11.0 Hz, 1H, CH_2Ph), 4.27 (d, *J* = 14.4 Hz, 1H, CH_2Ph), 4.22 (d, *J* = 11.7 Hz, 1H, CH_2Ph), 4.22 (d, *J* = 11.7 Hz, 1H, CH_2Ph), 4.08 (*pseudo* t, *J* = 9.6 Hz, 1H, H-4), 3.79 (d, *J* = 8.9 Hz, 1H, H-5), 3.69 (d, *J* = 10.3 Hz, 1H, H-6), 3.51 (d, *J* = 10.3 Hz, 1H,H-6'), 3.48 (d, *J* = 12.4 Hz, 1H, H-2). ^{13}C NMR (CD_2Cl_2, 150 MHz) δ 100.7 (C-1), 76.0 (C-5), 75.3 (C-3), 72.7 (*C*H_2Ph), 72.4 (*C*H_2Ph), 72.0 (C-4), 65.1 (C-6), 58.6 (C-2), 47.9 (*C*H_2Ph).

4. Glycosylation of glycosyl triflate with alcohols

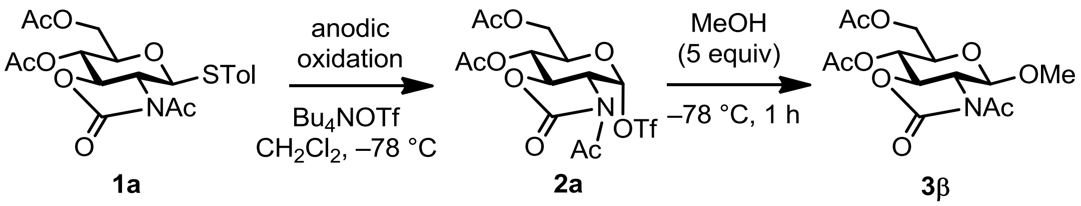

The anodic oxidation was carried out in an H-type divided cell (4G glass filter) equipped with a carbon felt anode (Nippon Carbon JF-20-P7, ca. 160 mg, dried at 250 °C/1 mmHg before use) and a platinum plate cathode (10 mm x 10 mm). In the anodic chamber were placed a thioglycoside **1a** (48.4 mg, 0.11 mmol) and 0.1 M Bu_4NOTf in CH_2Cl_2 (5.0 mL). In the cathodic chamber were placed trifluoromethanesulfonic acid (22 μL, 0.25 mmol) and 0.1 M Bu_4NOTf in CH_2Cl_2 (5.0 mL). The constant current electrolysis (4.0 mA) was carried out at −78 °C with magnetic stirring. After 1.6 F/mol of electricity was consumed, methanol (20 μL, 0.5 mmol) was added to the anodic chamber and the reaction mixture was stirred for an additional 1 h at −78 °C. Et_3N (0.1 mL) was added and the mixture was filtered through a short column (2 x 3 cm) of silica gel to remove Bu_4NOTf. The removal of solvent under reduced pressure afforded β-isomer of the corresponding methyl glycoside **3β** in 76% yield (29 mg, 0.084 mmol, >99% β-selectivity). **Methyl 2-*N*-acetyl-4,6-di-*O*-acetyl-2,3-*N*,*O*-carbonyl-2-deoxy-β-D-glucopyranoside** (**3β**). ^{1}H NMR ($CDCl_3$, 400 MHz) δ 5.10 (dd, *J* = 9.6, 3.6 Hz, 1H), 4.97 (d, *J* = 6.4 Hz, 1H), 4.50 (dd, *J* = 12.0, 4.8 Hz, 1H), 4.29 (dd, *J* = 12.8, 9.6 Hz, 1H), 4.24 (dd, *J* = 12.0, 7.2 Hz, 1H), 4.07 (ddd, *J* = 8.4, 6.4, 3.6 Hz, 1H), 3.91 (dd, *J* = 12.8, 6.4 Hz, 1H), 3.51

(s, 3H), 2.52 (s, 3H), 2.13 (s, 3H), 2.10 (s, 3H). ^{13}C NMR ($CDCl_3$, 100 MHz) δ 170.2, 170.1, 169.4, 152.8, 101.2, 77.5, 74.9, 70.1, 64.0, 60.5, 56.4, 24.6, 20.9, 20.8. HRMS (EI) *m/z* calcd for $C_{14}H_{19}NO_9$ $[M]^+$, 345.1060; found, 345.1057.

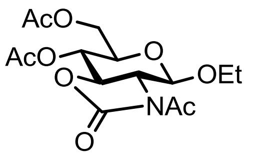

4β

Glycosylation of **2a** (43.5 mg, 0.099 mmol) with ethanol (29 μL, 0.50 mmol) afforded **ethyl 2-*N*-acetyl-4,6-di-*O*-acetyl-2,3-*N,O*-carbonyl-2-deoxy-β-D-glucopyranoside** (**4β**) in 71% yield (25 mg, 0.070 mmol, >99% β-selectivity). ^{1}H NMR ($CDCl_3$, 400 MHz) δ 5.10 (dd, *J* = 9.6, 3.6 Hz, 1H), 5.08 (d, *J* = 6.4 Hz, 1H), 4.51 (dd, *J* = 12.0, 4.8 Hz, 1H), 4.29 (dd, *J* = 12.4, 9.6 Hz, 1H), 4.26 (dd, *J* = 12.0, 7.2 Hz, 1H), 4.07 (ddd, *J* = 8.4, 4.8, 3.6 Hz, 1H), 3.95–3.86 (m, 2H), 3.62 (ddd, *J* = 16.0, 9.2, 7.2 Hz, 1H), 2.52 (s, 3H), 2.13 (s, 3H), 2.09 (s, 3H), 1.24 (t, *J* = 7.2 Hz, 3H). ^{13}C NMR ($CDCl_3$, 100 MHz) δ 170.14, 170.10, 169.4, 152.8, 99.9, 77.5, 74.9, 70.3, 64.9, 64.2, 60.8, 24.6, 20.9, 20.8, 14.9. HRMS (EI) *m/z* calcd for $C_{15}H_{21}NO_9$ $[M+H]^+$, 360.1289; found, 360.1292.

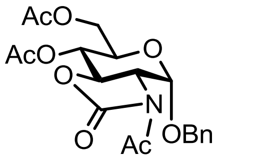

5α

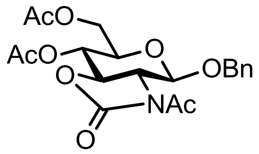

5β

Glycosylation of **2a** (44.5 mg, 0.102 mmol) with benzyl alcohol (52 μL, 0.50 mmol) afforded a mixture of **5α** and **5β** [2] in 89% yield (38 mg, 0.090 mmol, α/β = 9/91). **Benzyl-*N*-acetyl-4,6-di-*O*-acetyl-2,3-*N,O*-carbonyl-2-deoxy-α-D-glucopyranoside** (**5α**). ^{1}H NMR ($CDCl_3$, 600 MHz) δ 7.39–7.29 (m, 5H), 5.85 (d, *J* = 3.4 Hz, 1H), 5.29 (t, *J* = 10.3 Hz, 1H), 4.73 (d, *J* = 11.7 Hz, 1H), 4.69 (dd, *J* = 11.7, 10.3 Hz, 1H), 4.63 (d, *J* = 11.7 Hz, 1H), 4.22 (dd, *J* = 12.4, 4.8, 1H), 4.06 (dd, *J* = 12.4, 2.0 Hz, 1H), 3.90 (dd, *J* = 11.7, 2.8, 1H), 3.87–3.85 (ddd, *J* = 9.6, 4.8, 2.8 Hz, 1H), 2.49 (s, 3H), 2.11 (d, *J* = 6.2 Hz, 6H). ^{13}C NMR ($CDCl_3$, 150 MHz) δ 171.0, 170.6, 169.1, 152.6, 136.4, 128.6, 128.4, 128.1, 95.9, 74.3, 71.5, 70.1, 68.0, 61.5, 60.0, 23.6, 20.7, 20.6. HRMS (FAB) *m/z* calcd for $C_{20}H_{23}NO_9$ $[M+H]^+$, 422.1446; found, 422.1438.

Benzyl 2-*N*-acetyl-4,6-di-*O*-acetyl-2,3-*N,O*-carbonyl-2-deoxy-α-D-glucopyranoside (**5β**). ^{1}H NMR ($CDCl_3$, 400 MHz) δ 7.40–7.27 (m, 5H), 5.17 (d, *J* = 6.8 Hz, 1H), 5.11 (dd, *J* = 9.6, 3.6 Hz, 1H), 4.88 (d, *J* = 11.2 Hz, 1H), 4.65 (d, *J* = 11.2 Hz, 1H), 4.53 (dd, *J* = 12.0, 4.8 Hz, 1H), 4.29 (dd, *J* = 12.8, 9.6 Hz, 1H), 4.25 (dd, *J* = 11.6, 6.8 Hz, 1H), 4.09 (ddd, *J* = 7.2, 3.6, 1.2 Hz, 1H), 4.01 (dd, *J* = 12.8, 6.8 Hz, 1H), 2.52 (s, 3H), 2.13 (s, 3H), 2.00 (s, 3H). ^{13}C NMR ($CDCl_3$, 100 MHz) δ 170.1, 170.0, 169.3, 152.8, 136.3, 128.3, 128.0, 127.9, 99.5, 77.6, 74.9, 71.0, 70.2, 64.1, 60.5, 24.6, 20.83, 20.79.

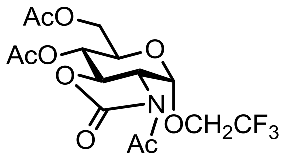

6α

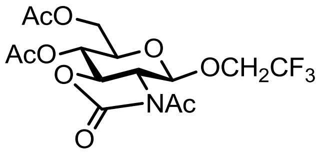

6β

Glycosylation of **2a** (43.7 mg, 0.10 mmol) with 2,2,2-trifluoroethanol (36 μL, 0.49 mmol) afforded a mixture of **6α** and **6β** in 82% yield (33.8 mg, 0.082 mmol, α/β = 15/85). **2',2',2'-Trifluoroethyl 2-*N*-acetyl-4,6-di-*O*-acetyl-2,3-*N,O*-carbonyl-2-deoxy-α-D-glucopyranoside** (**6α**). ^{1}H NMR ($CDCl_3$, 600 MHz) δ 5.79 (d, *J* = 2.8 Hz, 1H), 5.33 (t, *J* = 10.3 Hz, 1H), 4.64 (dd, *J* = 11.7, 10.3 Hz, 1H), 4.25 (dd, *J* = 12.4, 4.8 Hz, 1H), 4.18 (dd, *J* = 12.4, 2.0 Hz, 1H), 4.07–4.00 (m, 2H), 3.96–3.93 (ddd, *J* = 8.9, 4.1, 2.1 Hz, 1H), 3.90 (dd, *J* = 11.7, 2.8 Hz, 1H), 2.51 (s, 3H), 2.14 (s, 3H), 2.10 (s, 3H) ^{13}C NMR ($CDCl_3$, 150 MHz) δ 171.1, 170.5, 169.1, 152.3, 124.0 (q, *J* = 275.7 Hz), 96.5, 73.7, 70.9, 67.7, 66.3 (q, *J* = 34.5 Hz), 61.3, 59.5, 23.5, 20.6, 20.6. HRMS (EI) *m/z* calcd for $C_{15}H_{18}F_3NO_9$ $[M+NH_4]^+$, 431.1272; found, 431.1269.

2',2',2'-Trifluoroethyl 2-*N*-acetyl-4,6-di-*O*-acetyl-2,3-*N,O*-carbonyl-2-deoxy-β-D-glucopyranoside (**6β**); ^{1}H NMR ($CDCl_3$, 400 MHz) δ 5.22 (d, *J* = 6.4 Hz, 1H), 5.10 (dd, *J* = 9.6, 2.8 Hz, 1H), 4.55 (dd, *J* = 11.6, 4.4 Hz, 1H), 4.31 (dd, *J* = 12.8, 9.6 Hz, 1H), 4.23 (dd, *J* = 11.6, 7.6 Hz, 1H), 4.18–4.11 (m, 2H), 4.09–4.04 (m, 1H), 4.00 (dd, *J* = 12.8, 6.4 Hz, 1H), 2.53 (s, 3H), 2.14 (s, 3H), 2.10 (s, 3H). ^{13}C NMR ($CDCl_3$, 100 MHz) δ 170.3, 170.2, 169.7, 152.6, 123.3 (q, *J* = 276.1 Hz), 100.1, 78.5, 74.2, 70.1, 66.0 (q, *J* = 34.9 Hz), 64.2, 60.0, 24.3, 20.6. HRMS (EI) *m/z* calcd for $C_{15}H_{18}F_3NO_9$ $[M]^+$, 413.0934; found, 414.1006.

5. Electrochemical glycosylation in the presence of glycosyl acceptor

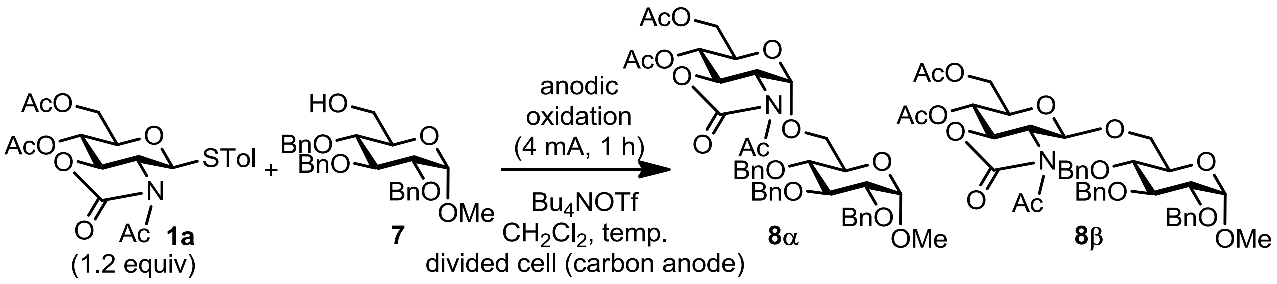

The anodic oxidation was carried out in an H-type divided cell (4G glass filter) equipped with a carbon felt anode (Nippon Carbon JF-20-P7, ca. 160 mg, dried at 250 °C/1 mmHg before use) and a platinum plate cathode (10 mm x 10 mm). In the anodic chamber were placed a thioglycoside **1a** (52.4 mg, 0.12 mmol), glycosyl acceptor **3** (46.2 mg, 0.099 mmol), and 0.1 M Bu_4NOTf in CH_2Cl_2 (5.0 mL). In the cathodic chamber were placed trifluoromethanesulfonic acid (18 μL, 0.20 mmol) and 0.1 M Bu_4NOTf in CH_2Cl_2 (5.0 mL). The constant current electrolysis (4.0 mA) was carried out at −78 °C with magnetic stirring. After 1.0 F/mol of electricity was consumed, Et_3N (0.5 mL) was added and the reaction mixture was filtered through a short column (2 x 3 cm) of silica gel to remove Bu_4NOTf. The removal of solvent under reduced pressure afforded the β-isomer of the corresponding disaccharide **8α** [4]/**8β** [4] in 36% NMR yield (**8α**/**8β** 46:54) (Table 3, entry 1).

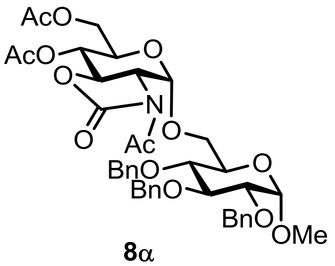

8α

Methyl 2-*N*-acetyl-4,6-di-*O*-acetyl-2,3-*N*,*O*-carbonyl-2-deoxy-α-D-glucopyranosyl-(1→6)-2,3,4-tri-*O*-benzyl-α-D-glucopyranoside (8α). ^{1}H NMR ($CDCl_3$, 400 MHz) δ 7.36–7.22 (m, 15H), 5.76 (d, *J* = 2.4 Hz, 1H), 5.25 (dd, *J* = 10.0, 9.6 Hz, 1H), 5.00 (d, *J* = 11.2 Hz, 1H), 4.86 (d, *J* = 11.6 Hz, 1H), 4.79 (d, *J* = 10.4 Hz, 1H), 4.76 (d, *J* = 11.6 Hz, 1H), 4.67 (d, *J* = 12.0 Hz, 1H), 4.58 (d, *J* = 3.2 Hz, 1H), 4.56 (d, *J* = 10.8 Hz, 1H), 4.49 (dd, *J* = 12.0, 10.4 Hz, 1H), 4.14 (dd, *J* = 12.4, 4.0 Hz, 1H), 4.09 (dd, *J* = 12.4, 2.4 Hz, 1H), 3.98 (dd, *J* = 9.6, 8.8 Hz, 1H), 3.85 (ddd, *J* = 9.6, 4.0, 2.4 Hz, 1H), 3.80 (dd, *J* = 12.4, 2.4 Hz, 1H), 3.78 (d, *J* = 3.6 Hz, 1H), 3.71 (*pseudo* dt, *J* = 10.0, 3.6 Hz, 1H), 3.51 (dd, *J* = 9.6, 3.6 Hz, 1H), 3.35 (s, 3H), 3.30 (dd, *J* = 10.0, 8.8 Hz, 1H), 2.40 (s, 3H), 2.11 (s, 3H), 2.02 (s, 3H). ^{13}C NMR ($CDCl_3$, 100 MHz) δ 170.7, 170.3, 168.9, 152.5, 138.5, 137.8, 137.7, 128.3, 128.28, 128.25, 127.9, 127.8, 127.78, 127.75, 127.70, 127.5, 97.7, 95.4, 81.9, 79.7, 77.0, 75.6, 74.6, 73.9, 73.2, 69.9 (2C), 68.0, 66.9, 61.5, 59.9, 55.2, 23.7, 20.79, 20.75.

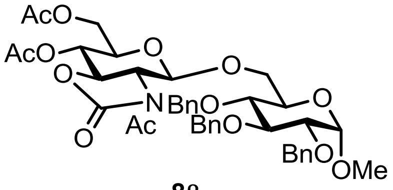

8β

Methyl 2-*N*-acetyl-4,6-di-*O*-acetyl-2,3-*N*,*O*-carbonyl-2-deoxy-β-D-glucopyranosyl-(1→6)-2,3,4-tri-*O*-benzyl-α-D-glucopyranoside (8β). ^{1}H NMR ($CDCl_3$, 400 MHz) δ 7.34–7.24 (m, 15H), 5.15 (dd, *J* = 9.6, 4.4 Hz, 1H), 4.99 (d, *J* = 11.2 Hz, 1H), 4.96 (d, *J* = 6.8 Hz, 1H), 4.89 (d, *J* = 11.2 Hz, 1H), 4.81 (d, *J* = 10.8 Hz, 1H), 4.78 (d, *J* = 12.4 Hz, 1H), 4.72 (d, *J* = 11.2 Hz, 1H), 4.67 (d, *J* = 12.4 Hz, 1H), 4.64 (d, *J* = 3.6 Hz, 1H), 4.39 (dd, *J* = 12.0, 4.8 Hz, 1H), 4.26 (dd, *J* = 12.0, 6.0 Hz, 1H), 4.21 (dd, *J* = 12.4, 9.6 Hz, 1H), 4.02–3.95 (m, 4H), 3.79 (dd, *J* = 10.8, 4.4 Hz, 1H), 3.74 (ddd, *J* = 9.6, 4.0, 2.0 Hz, 1H), 3.58 (dd, *J* = 9.6, 8.4 Hz, 1H), 3.54 (dd, *J* = 9.6, 3.6 Hz, 1H), 3.37 (s, 3H), 2.46 (s, 3H), 2.12 (s, 3H), 2.01 (s, 3H). ^{13}C NMR ($CDCl_3$, 100 MHz) δ 170.3, 170.0, 169.4, 153.1, 138.8, 138.7, 138.2, 128.4, 128.3, 128.1, 127.9, 127.8, 127.6, 127.5, 100.3, 97.9, 82.1, 79.9, 77.3, 77.1, 75.6, 75.2, 74.6, 73.2, 69.8, 69.6, 64.1, 60.4, 55.2, 24.5, 20.8, 20.7.

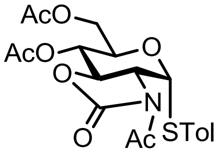

9

Electrochemical glycosylation of **1a** (52.6 mg, 0.120 mmol) with glycosyl acceptor **7** (46.4 mg, 0.100 mmol) at 0 °C afforded disaccharide **8α** in 59% NMR yield together with ***p*-methylphenyl 2-*N*-acetyl-4,6-di-*O*-acetyl-2,3-*N*,*O*-carbonyl-2-deoxy-1-thio-α-D-glucopyranoside** (**9**) in 21% yield (11.3 mg, 0.025 mmol) after purification by silica gel chromatography (Table 3, entry 4). ^{1}H NMR ($CDCl_3$, 400 MHz) δ 7.30 (m, 2H), 7.13 (d, *J* = 8.0 Hz, 2H), 6.12 (d, *J* = 4.0, 1H), 5.30 (dd, *J* = 10.0, 9.2 Hz, 1H), 4.47 (dd, *J* = 12.0, 10.0 Hz, 1H),

4.38–4.43 (m, 1H), 4.33 (dd, *J* = 12.0, 4.8 Hz, 1H), 4.16 (m, 2H) 2.55 (s, 3H), 2.34 (s, 3H), 2.15 (s, 3H), 2.08 (s, 3H). ^{13}C NMR ($CDCl_3$, 100 MHz) δ 171.0, 170.5, 169.2, 152.5, 138.7, 133.2, 130.1, 128.0, 86.5, 75.9, 70.2, 67.9, 61.7, 59.9, 23.8, 21.2, 20.7, 20.6. HRMS (FAB) *m/z* calcd for $C_{20}H_{23}NO_8S$ $[M+H]^+$, 438.1217; found, 438.1212.

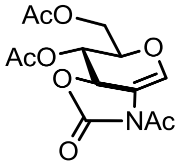

10

Electrochemical glycosylation of **1a** (52.5 mg, 0.12 mmol) with glycosyl acceptor **7** (46.2 mg, 0.10 mmol) at −78 °C in the presence of DTBMP afforded glucal **10** in 24% NMR yield as a major byproduct derived from **1a** (Table 3, entry 2). **2-*N*-acetyl-4,6-di-*O*-acetyl-2,3-*N,O*-carbonyl -D-glucal** (**10**). ^{1}H NMR ($CDCl_3$, 400 MHz) δ 7.31 (d, *J* = 2.4 Hz, 1H), 5.42 (dd, *J* = 10.4, 8.4 Hz, 1H), 5.08 (dd, *J* = 8.0, 2.4 Hz, 1H), 4.33 (dd, *J* = 12.4, 4.0 Hz, 1H), 4.23 (dd, *J* = 12.4, 2.0 Hz, 1H), 4.15 (ddd, *J* = 10.0, 4.0, 2.0 Hz, 1H), 2.56 (s, 3H), 2.15 (s, 3H), 2.09 (s, 3H). ^{13}C NMR ($CDCl_3$, 100 MHz) δ 170.2, 168.6, 168.3, 151.8, 132.5, 111.6. 74.5, 72.9, 66.0, 61.3, 24.1, 20.8. HRMS (EI) *m/z* calcd for $C_{13}H_{15}NO_8$, 313.0798; found, 313.0800.

6. Triflic acid mediated isomerization of β-isomer to α-isomer

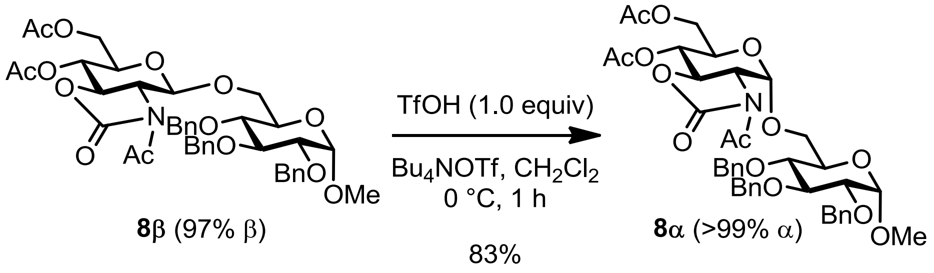

The crude mixture of electrochemical glycosylation **8α**/**8β** (0.094 mmol, **8α/8β** 3:97) was treated with TfOH (0.094 mmol, 8 μL) and 0.1 M Bu_4NOTf in CH_2Cl_2 (5.0 mL) at 0 °C for 1 h. Then Et_3N (0.5 mL) was added and the removal of solvent under reduced pressure afforded the α-isomer of the corresponding disaccharide **8α** as a single product in 83% NMR yield.

7. References

1. Lin, S.-C.; Chao, C.-S.; Chang, C.-C.; Mong, K.-K. T. *Tetrahedron Lett.* **2010**, *51*, 1910.
2. Wei, P.; Kerns, R. J. *J. Org. Chem*. **2005**, *70,* 4195.
3. Bernet, B.; Vasella, A. *Helv. Chim. Acta* **1979**, *64*, 1990.
4. Geng, Y.; Zhang, L.-H.; Ye, X.-S. *Chem. Commun.* **2008**, 597.

C:\WINNMR98\COMMON_DEFAULT.ALS

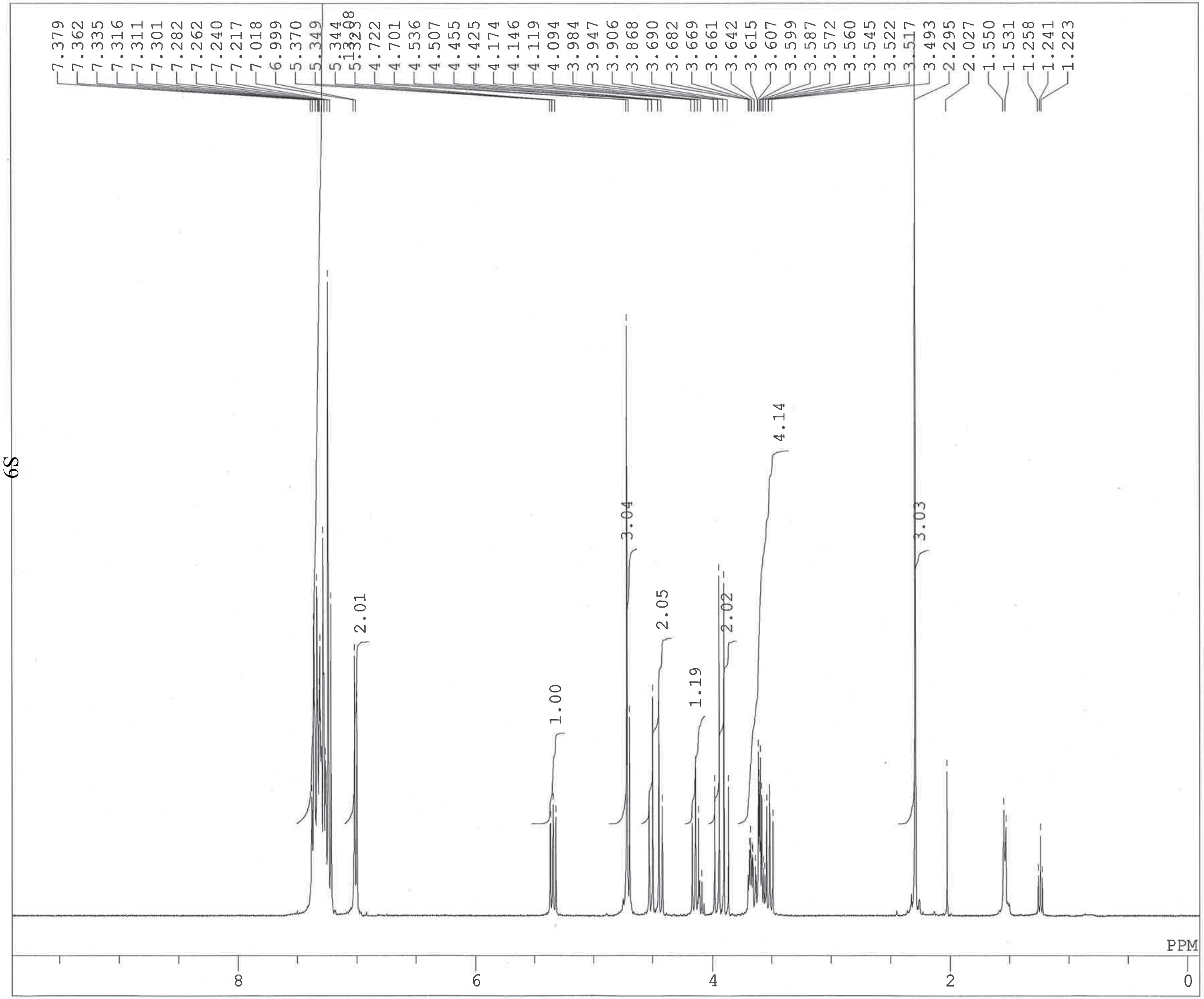

DFILE C:\WINNMR98\COMMON_DEFAULT.A
COMNT
DATIM Sat Jan 07 20:16:22 2012
OBNUC 1H
EXMOD NON
OBFRQ 399.65 MHz
OBSET 133.90 KHz
OBFIN 92.5 Hz
POINT 32768
FREQU 8000.0 Hz
SCANS 16
ACQTM 4.096 sec
PD 2.901 sec
PW1 11.5 us
IRNUC 1H
CTEMP 22.5 c
SLVNT CDCL3
EXREF 7.24 ppm
BF 0.12 Hz
RGAIN 19

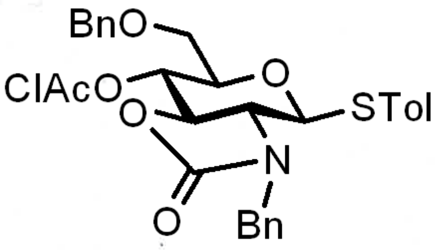

1b

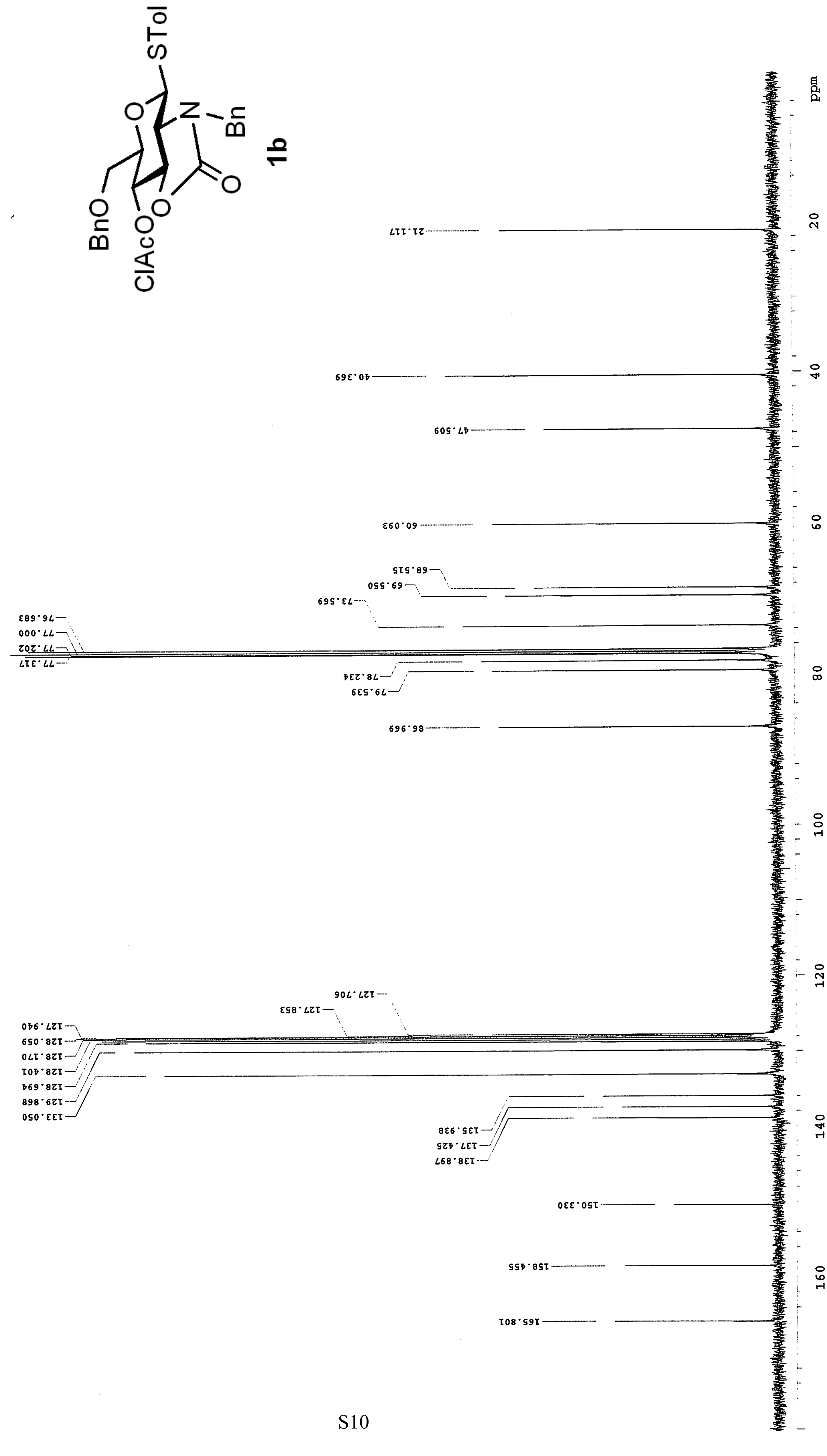
BnO
ClAcO
O
O
O
N
Bn
STol
1b
21.117
40.369
47.509
60.093
68.515
69.550
73.569
76.683
77.000
77.202
77.317
78.234
79.539
86.969
127.706
127.853
127.940
128.059
128.170
128.401
128.694
129.868
133.050
135.938
137.425
138.897
150.330
158.455
165.801
ppm
20
40
60
80
100
120
140
160

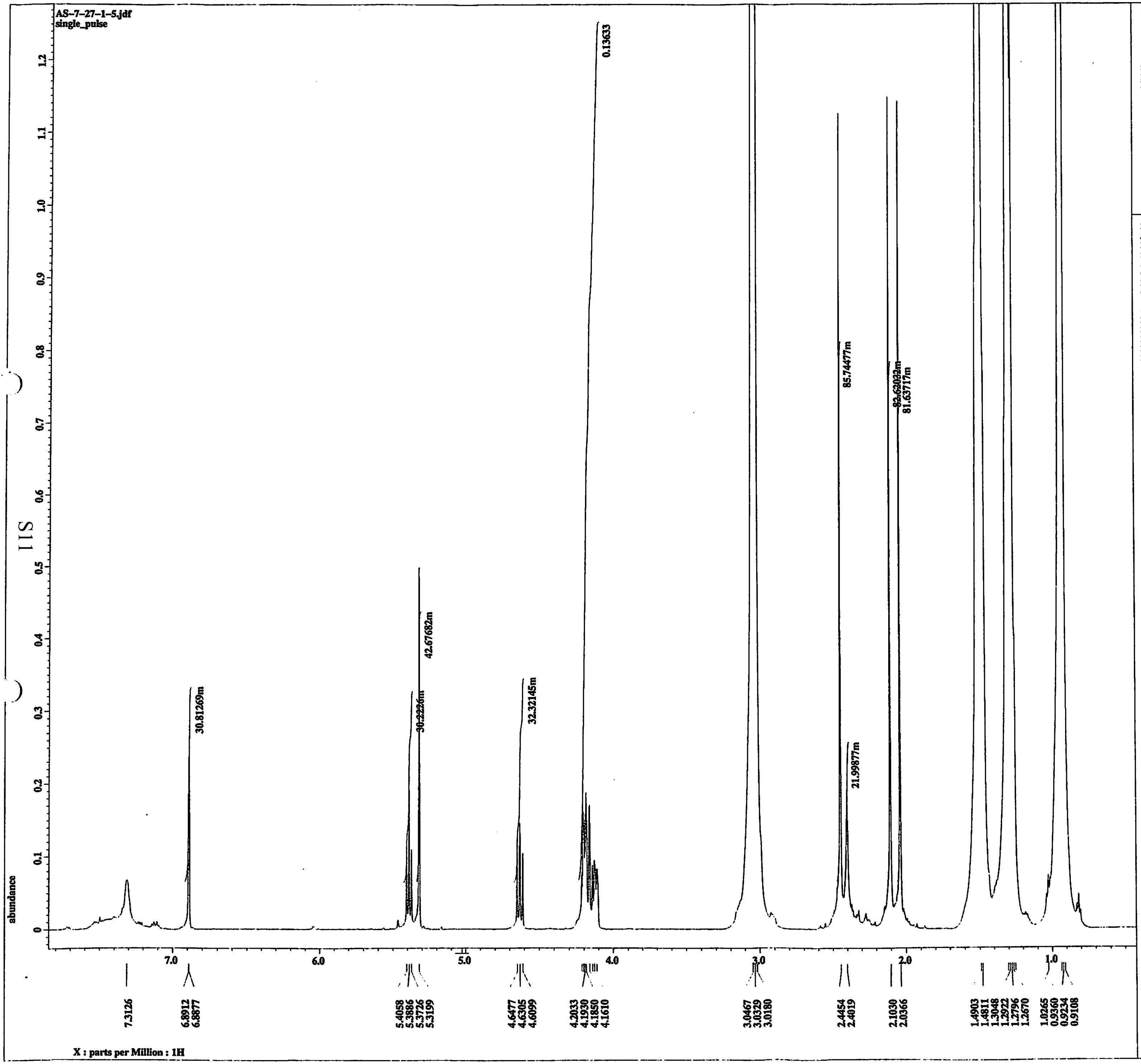

---- PROCESSING PARAMETERS ----
dc_balance : 0 : FALSE
sexp : 0.2[Hz] : 0.0[s]
trapezoid3 : 0[%] : 80[%] : 100[%]
zerofill : 1
fft : 1 : TRUE : TRUE
machinephase
ppm
base_correct : None : 0 : Smooth

Derived from: AS-7-27-1-1.jdf

Filename	= AS-7-27-1-5.jdf
Author	= delta
Experiment	= single_pulse.ex2
Sample_id	= S#480407
Solvent	= METHYLENE-CHLORI
Creation_time	= 28-SEP-2007 13:23:33
Revision_time	= 28-SEP-2007 15:06:30
Current_time	= 28-SEP-2007 15:06:55
Content	= single_pulse
Data_format	= 1D REAL
Dim_size	= 13107
Dim_title	= 1H
Dim_units	= [ppm]
Dimensions	= X
Site	= ECA600
Spectrometer	= JNM-ECA600
Field_strength	= 14.09636928[T] (600[M
X_acq_duration	= 1.4548992[s]
X_domain	= 1H
X_freq	= 600.1723046[MHz]
X_offset	= 5[ppm]
X_points	= 16384
X_prescans	= 1
X_resolution	= 0.68733284[Hz]
X_sweep	= 11.26126126[kHz]
Irr_domain	= 1H
Irr_freq	= 600.1723046[MHz]
Irr_offset	= 5[ppm]
Tri_domain	= 1H
Tri_freq	= 600.1723046[MHz]
Tri_offset	= 5[ppm]
Clipped	= FALSE
Mod_return	= 1
Scans	= 16
Total_scans	= 16
X_90_width	= 13.5[us]
X_acq_time	= 1.4548992[s]
X_angle	= 45[deg]
X_atn	= 7.8[dB]
X_pulse	= 6.75[us]
Irr_mode	= Off
Tri_mode	= Off
Dante_presat	= FALSE
Initial_wait	= 1[s]
Recvr_gain	= 40
Relaxation_delay	= 5[s]
Repetition_time	= 6.4548992[s]
Temp_get	= -80[dC]

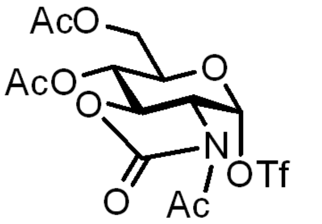

2a

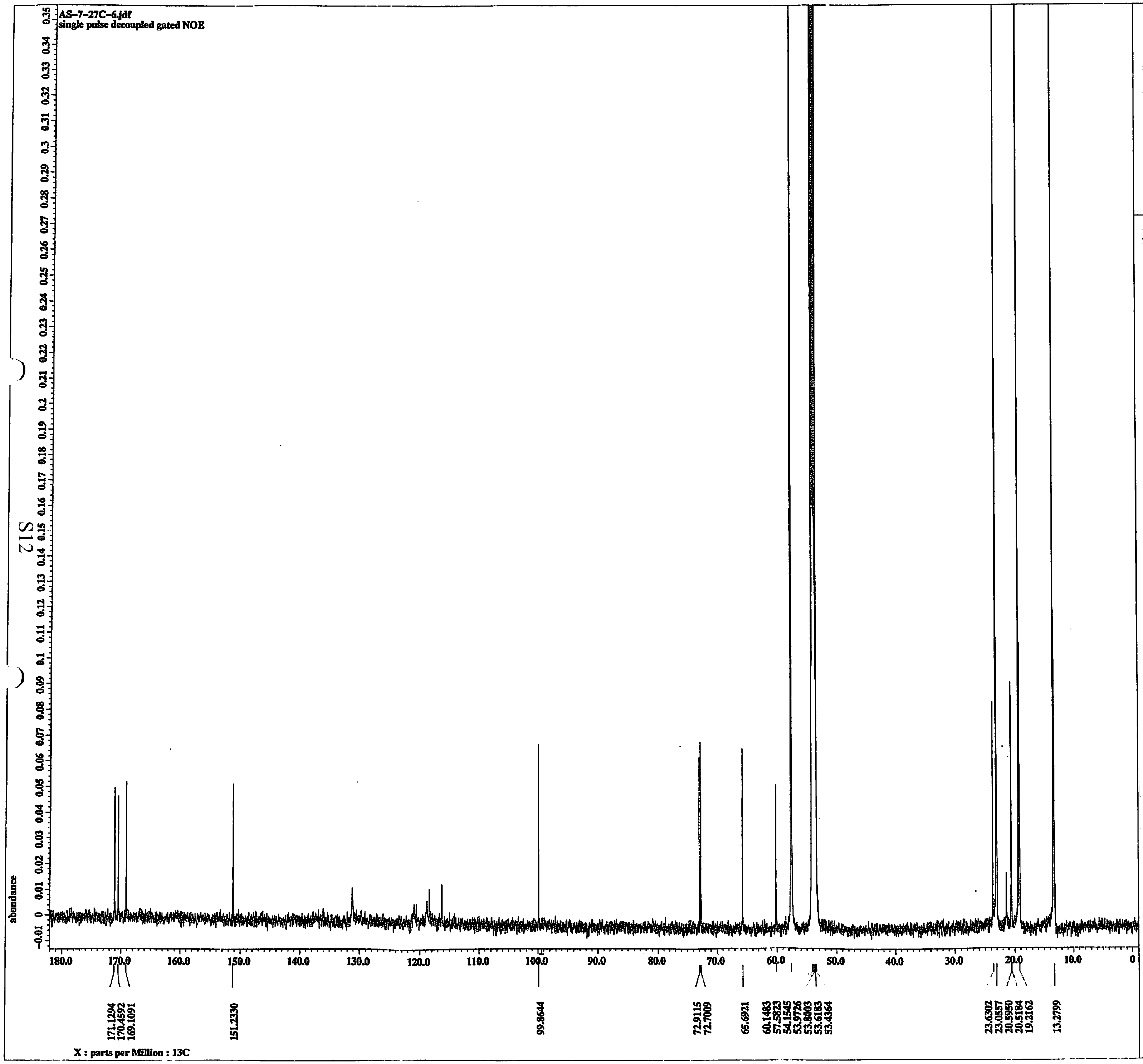


```
---- PROCESSING PARAMETERS ----
dc_balance : 0 : FALSE
sexp : 2.0[Hz] : 0.0[s]
trapezoid3 : 0[%] : 80[%] : 100[%]
zerofill : 1
fft : 1 : TRUE : TRUE
machinephase
ppm

Derived from: AS-7-27C-1.jdf
```

```
Filename           = AS-7-27C-6.jdf
Author             = delta
Experiment         = single_pulse_dec
Sample_id          = S#506417
Solvent            = METHYLENE-CHLORI
Creation_time      = 28-SEP-2007 17:21:55
Revision_time      = 28-SEP-2007 17:27:43
Current_time       = 28-SEP-2007 17:28:37

Content            = single pulse decouple
Data_format        = 1D COMPLEX
Dim_size           = 26214
Dim_title          = 13C
Dim_units          = [ppm]
Dimensions         = X
Site               = ECA600
Spectrometer       = JNM-ECA600

Field_strength     = 14.09636928[T] (600[M
X_acq_duration     = 0.69206016[s]
X_domain           = 13C
X_freq             = 150.91343039[MHz]
X_offset           = 100[ppm]
X_points           = 32768
X_prescans         = 4
X_resolution       = 1.44496109[Hz]
X_sweep            = 47.34848485[kHz]
Irr_domain         = 1H
Irr_freq           = 600.1723046[MHz]
Irr_offset         = 5[ppm]
Clipped            = FALSE
Mod_return         = 1
Scans              = 4356
Total_scans        = 4356

X_90_width         = 11.2[us]
X_acq_time         = 0.69206016[s]
X_angle            = 30[deg]
X_atn              = 8[dB]
X_pulse            = 3.73333333[us]
Irr_atn_dec        = 21.2[dB]
Irr_atn_noe        = 21.2[dB]
Irr_noise          = WALTZ
Decoupling         = TRUE
Initial_wait       = 1[s]
Noe                = TRUE
Noe_time           = 2[s]
Recvr_gain         = 60
Relaxation_delay   = 2[s]
Repetition_time    = 2.69206016[s]
Temp_get           = -80[dC]
```

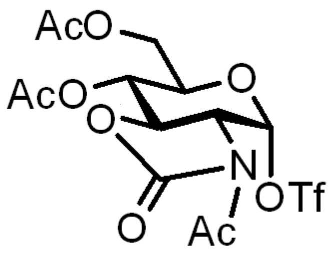

2a

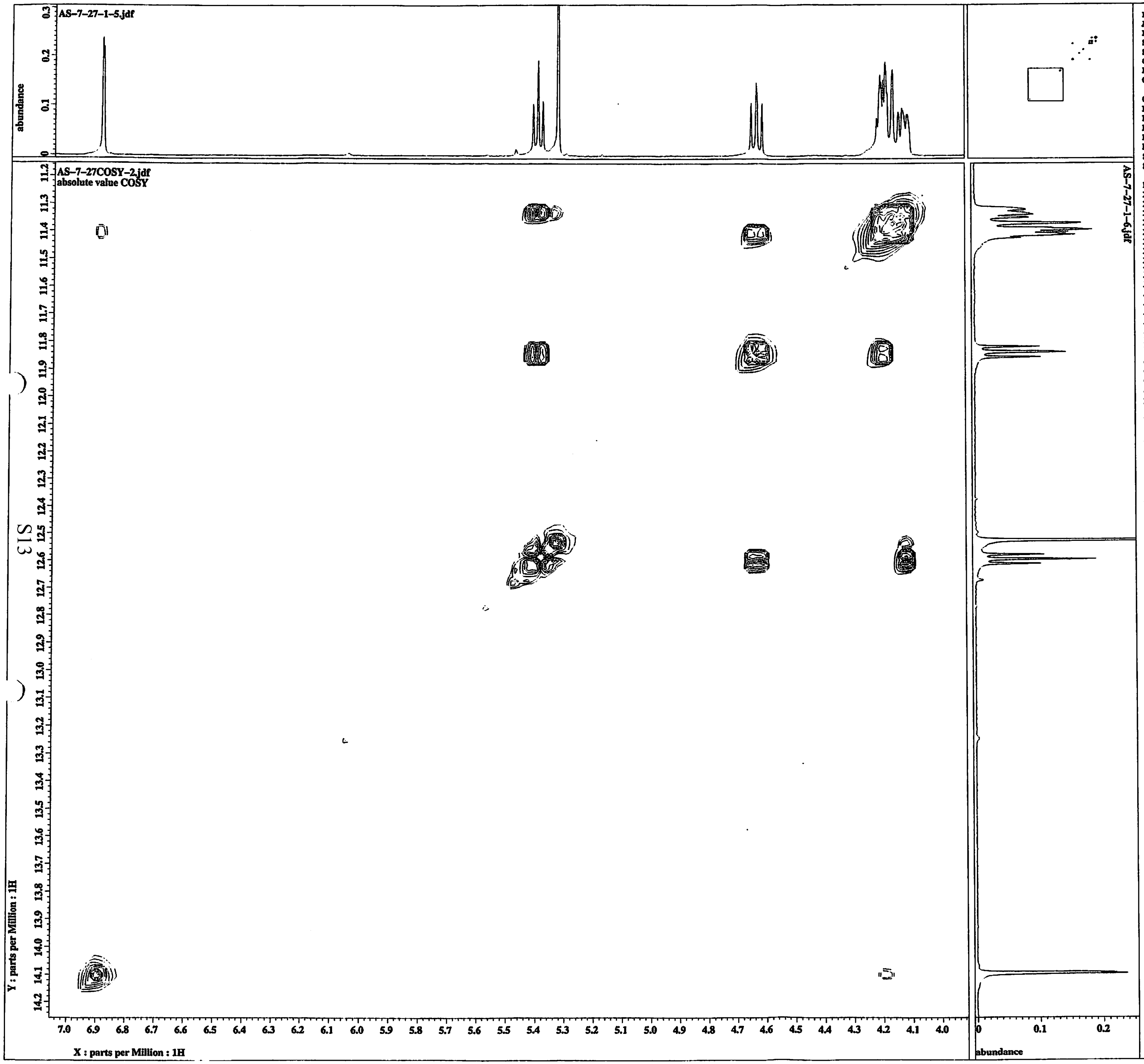


```
Filename             = AS-7-27COSY-2.jdf
Author               = delta
Experiment           = cosy.ex2
Sample_id            = S#485913
Solvent              = METHYLENE-CHLORI
Creation_time        = 28-SEP-2007 13:58:36
Revision_time        = 28-SEP-2007 15:13:00
Current_time         = 28-SEP-2007 15:15:30

Content              = absolute value COSY
Data_format          = 2D REAL REAL
Dim_size             = 1024, 1024
Dim_title            = 1H 1H
Dim_units            = [ppm] [ppm]
Dimensions           = X Y
Site                 = ECA600
Spectrometer         = JNM-ECA600

Field_strength       = 14.09636928[T] (600[M
X_acq_duration       = 0.113664[s]
X_domain             = 1H
X_freq               = 600.1723046[MHz]
X_offset             = 5[ppm]
X_points             = 1280
X_prescans           = 4
X_resolution         = 8.79786036[Hz]
X_sweep              = 11.26126126[kHz]
Y_domain             = 1H
Y_freq               = 600.1723046[MHz]
Y_offset             = 5[ppm]
Y_points             = 256
Y_prescans           = 0
Y_resolution         = 35.17242932[Hz]
Y_sweep              = 9.00414191[kHz]
Irr_domain           = 1H
Irr_freq             = 600.1723046[MHz]
Irr_offset           = 5[ppm]
Tri_domain           = 1H
Tri_freq             = 600.1723046[MHz]
Tri_offset           = 5[ppm]
Clipped              = TRUE
Mod_return           = 1
Scans                = 4
Total_scans          = 1024

X_90_width           = 13.5[us]
X_acq_time           = 0.113664[s]
X_atn                = 7.8[dB]
X_pulse              = 13.5[us]
Y_acq_time           = 28.43136[ms]
Irr_mode             = Off
Tri_mode             = Off
Dante_presat         = FALSE
Delta                = 0[ms]
Initial_wait         = 1[s]
Pulse_1              = 13.5[us]
Pulse_2              = 13.5[us]
Pulse_angle_1        = 90[deg]
Pulse_angle_2        = 90[deg]
Recvr_gain           = 42
Relaxation_delay     = 1.5[s]
Repetition_time      = 1.613664[s]
T1                   = 1[us]
Temp_get             = -80[dC]
```

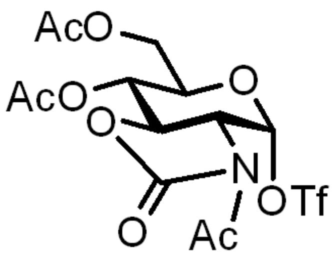

2a

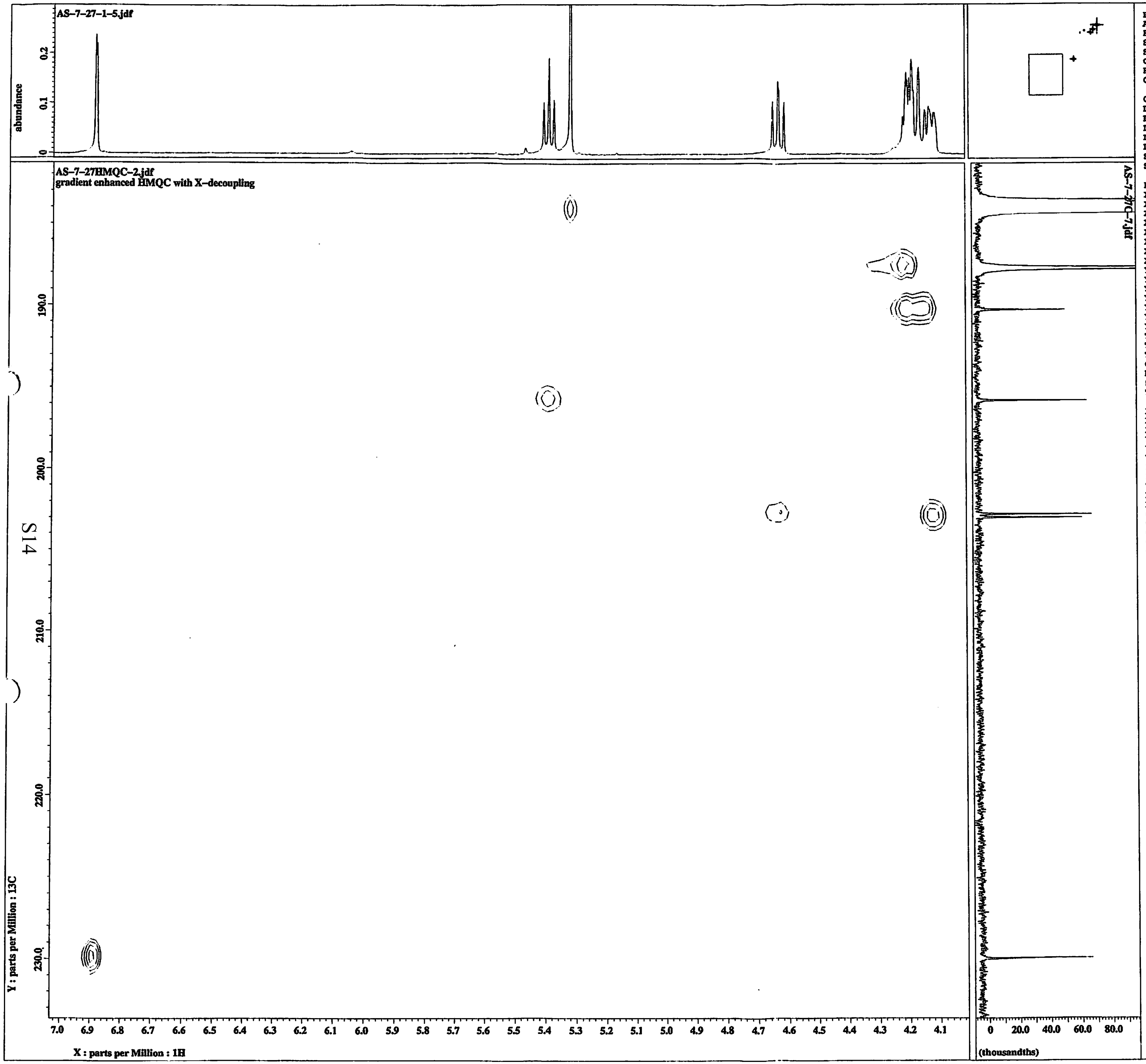

Filename	= AS-7-27HMQC-2.jdf
Author	= delta
Experiment	= hmqc_pfg.ex2
Sample_id	= S#626074
Solvent	= METHYLENE-CHLORI
Creation_time	= 28-SEP-2007 17:51:35
Revision_time	= 28-SEP-2007 17:54:51
Current_time	= 28-SEP-2007 18:13:15
Content	= gradient enhanced HMQ
Data_format	= 2D REAL REAL
Dim_size	= 819, 512
Dim_title	= 1H 13C
Dim_units	= [ppm] [ppm]
Dimensions	= X Y
Site	= ECA600
Spectrometer	= JNM-ECA600
Field_strength	= 14.09636928[T] (600[M
X_acq_duration	= 90.9312[ms]
X_domain	= 1H
X_freq	= 600.1723046[MHz]
X_offset	= 5[ppm]
X_points	= 1024
X_prescans	= 4
X_resolution	= 10.99732545[Hz]
X_sweep	= 11.26126126[kHz]
Y_domain	= 13C
Y_freq	= 150.91343039[MHz]
Y_offset	= 85[ppm]
Y_points	= 256
Y_prescans	= 0
Y_resolution	= 117.94233092[Hz]
Y_sweep	= 30.19323671[kHz]
Tri_domain	= 1H
Tri_freq	= 600.1723046[MHz]
Tri_offset	= 5[ppm]
Clipped	= FALSE
Mod_return	= 1
Scans	= 4
Total_scans	= 1024
X_acq_time	= 90.9312[ms]
X_atn	= 7.8[dB]
X_pulse	= 13.5[us]
Y_acq_time	= 8.47872[ms]
Y_atn	= 8[dB]
Y_pulse	= 11.2[us]
Irr_atn_dec	= 24.4[dB]
Irr_noise	= MPF8
Tri_mode	= Off
Dante_presat	= FALSE
Decoupling	= TRUE
Grad_1	= 1[ms]
Grad_1_amp	= 60[%]
Grad_2	= 1[ms]
Grad_2_amp	= 60[%]
Grad_3	= 1[ms]
Grad_3_amp	= 30.18108652[%]
Grad_recover	= 0.1[ms]
Grad_selection	= 13C = 1.988:1.98
Grad_shape	= SINE
Initial_wait	= 1[s]
J_constant	= 140[Hz]
Recvr_gain	= 84
Refocus_comp	= 2.57142857[ms]
Relaxation_delay	= 1.5[s]
Repetition_time	= 1.5909312[s]
T1	= 1[us]
Temp1	= 1.988
Temp_get	= -80[dC]

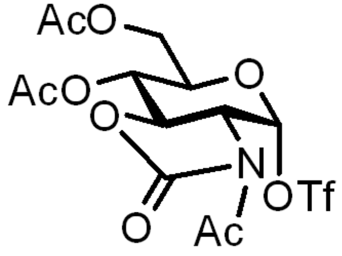

2a

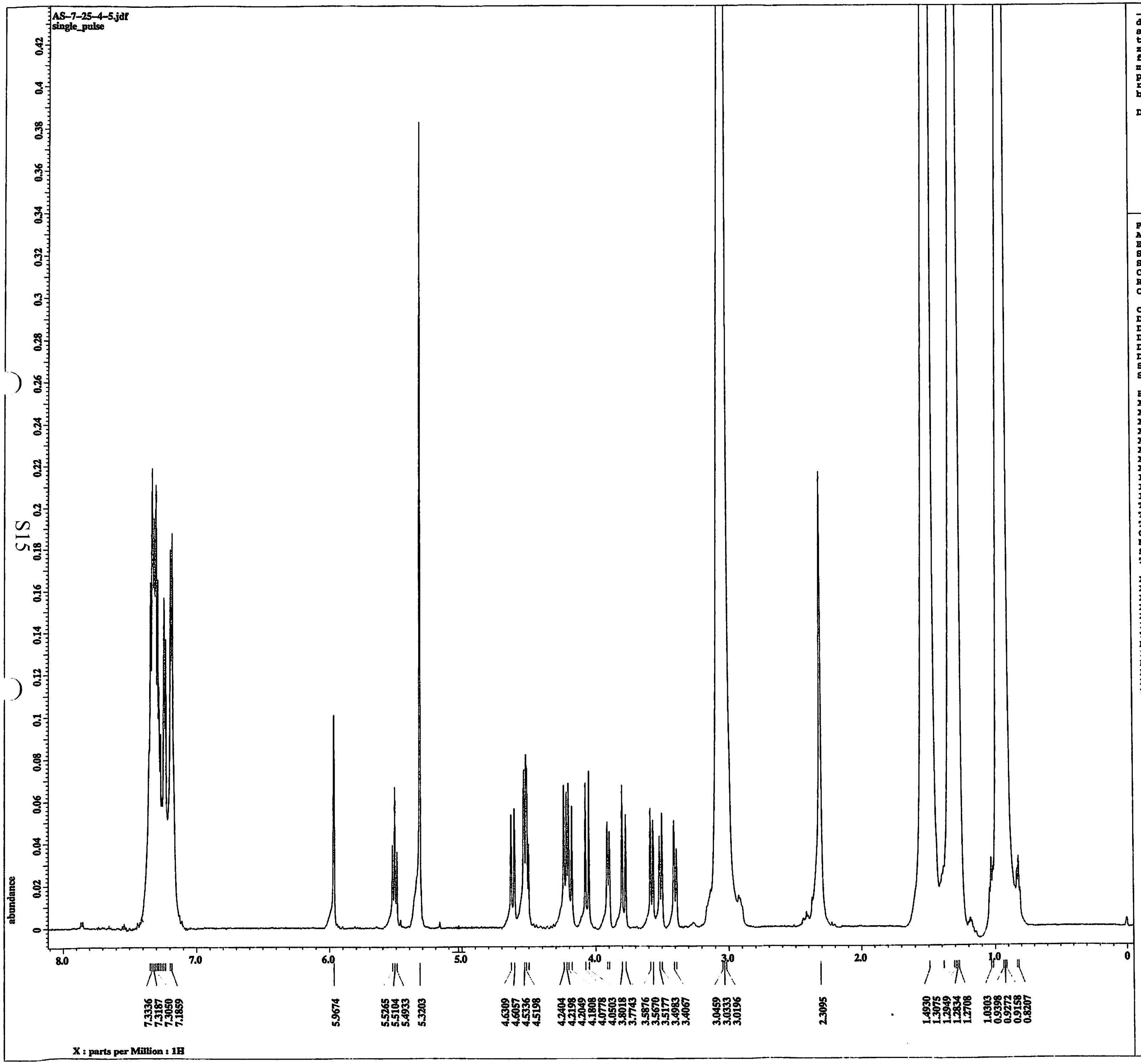

---- PROCESSING PARAMETERS ----
dc_balance : 0 : FALSE
sexp : 0.2[Hz] : 0.0[s]
trapezoid3 : 0[%] : 80[%] : 100[%]
zerofill : 1
fft : 1 : TRUE : TRUE
machinephase
ppm
reference : 5.294[ppm] : 5.32[ppm]
base_correct : None : 0 : Smooth

Derived from: AS-7-25-4-1.jdf

Filename = AS-7-25-4-5.jdf
Author = delta
Experiment = single_pulse.ex2
Sample_id = S#706885
Solvent = METHYLENE-CHLORI
Creation_time = 26-SEP-2007 19:41:00
Revision_time = 27-SEP-2007 11:56:22
Current_time = 27-SEP-2007 11:56:48

Content = single_pulse
Data_format = 1D REAL
Dim_size = 13107
Dim_title = 1H
Dim_units = [ppm]
Dimensions = X
Site = ECA600
Spectrometer = JNM-ECA600

Field_strength = 14.09636928[T] (600[M
X_acq_duration = 1.4548992[s]
X_domain = 1H
X_freq = 600.1723046[MHz]
X_offset = 5[ppm]
X_points = 16384
X_prescans = 1
X_resolution = 0.68733284[Hz]
X_sweep = 11.26126126[kHz]
Irr_domain = 1H
Irr_freq = 600.1723046[MHz]
Irr_offset = 5[ppm]
Tri_domain = 1H
Tri_freq = 600.1723046[MHz]
Tri_offset = 5[ppm]
Clipped = FALSE
Mod_return = 1
Scans = 16
Total_scans = 16

X_90_width = 13.5[us]
X_acq_time = 1.4548992[s]
X_angle = 45[deg]
X_atn = 7.8[dB]
X_pulse = 6.75[us]
Irr_mode = Off
Tri_mode = Off
Dante_presat = FALSE
Initial_wait = 1[s]
Recvr_gain = 38
Relaxation_delay = 5[s]
Repetition_time = 6.4548992[s]
Temp_get = -80[dC]

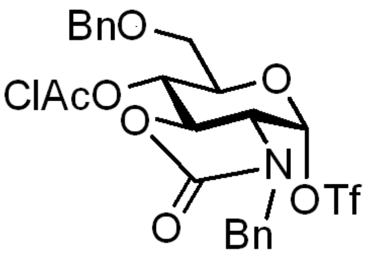

2b

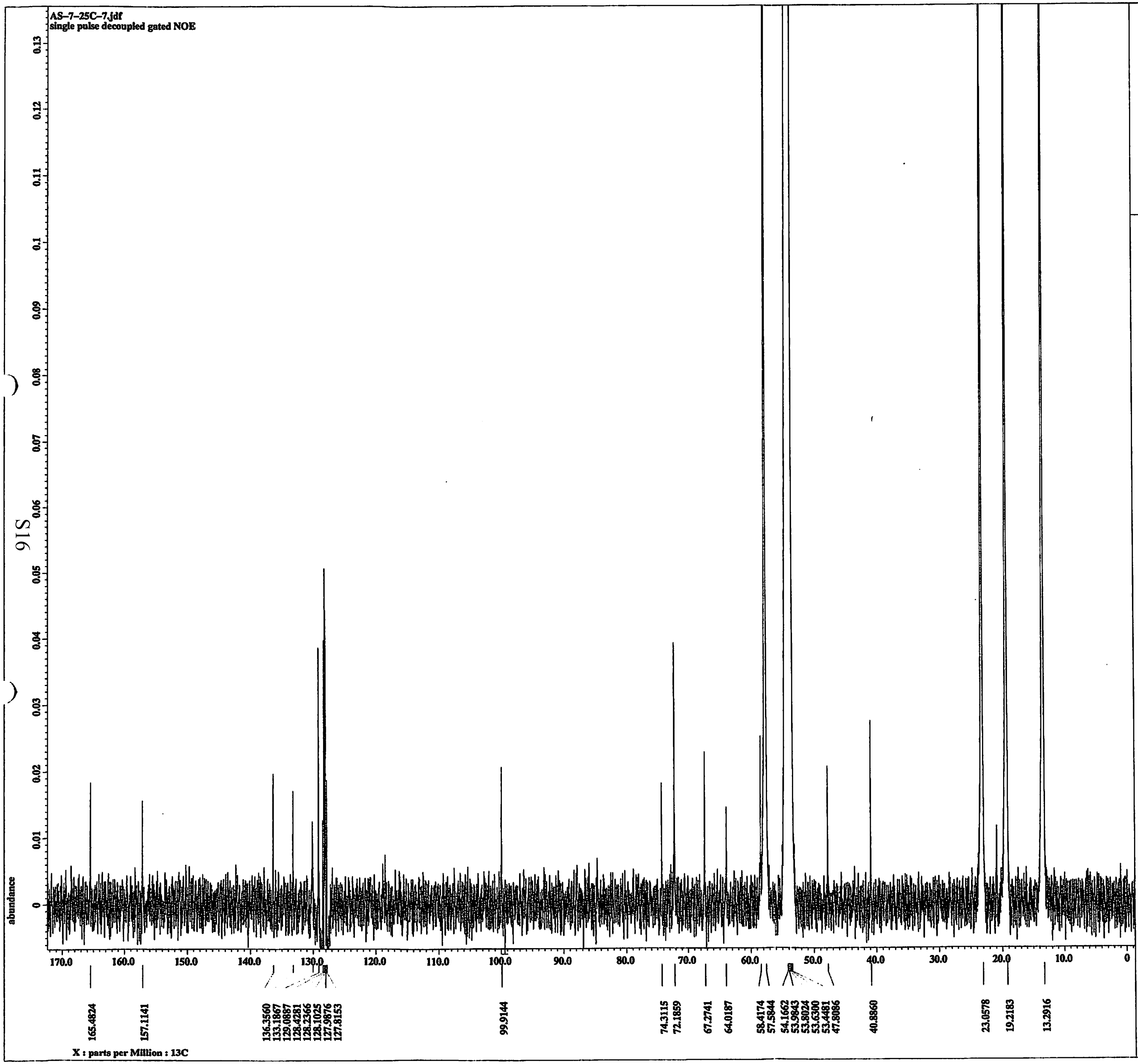


```
---- PROCESSING PARAMETERS ----
dc_balance : 0 : FALSE
sexp : 2.0[Hz] : 0.0[s]
trapezoid3 : 0[%] : 80[%] : 100[%]
zerofill : 1
fft : 1 : TRUE : TRUE
machinephase
ppm
reference : 54.395[ppm] : 53.802[ppm]
base_correct : None : 0 : Smooth

Derived from: AS-7-25C-1.jdf
```

```
Filename            = AS-7-25C-7.jdf
Author              = delta
Experiment          = single_pulse_dec
Sample_id           = S#514090
Solvent             = METHYLENE-CHLORI
Creation_time       = 26-SEP-2007 15:42:17
Revision_time       = 26-SEP-2007 16:59:51
Current_time        = 26-SEP-2007 16:59:57

Content             = single pulse decouple
Data_format         = 1D REAL
Dim_size            = 26214
Dim_title           = 13C
Dim_units           = [ppm]
Dimensions          = X
Site                = ECA600
Spectrometer        = JNM-ECA600

Field_strength      = 14.09636928[T] (600[M
X_acq_duration      = 0.69206016[s]
X_domain            = 13C
X_freq              = 150.91343039[MHz]
X_offset            = 100[ppm]
X_points            = 32768
X_prescans          = 4
X_resolution        = 1.44496109[Hz]
X_sweep             = 47.34848485[kHz]
Irr_domain          = 1H
Irr_freq            = 600.1723046[MHz]
Irr_offset          = 5[ppm]
Clipped             = TRUE
Mod_return          = 1
Scans               = 1883
Total_scans         = 1883

X_90_width          = 11.2[us]
X_acq_time          = 0.69206016[s]
X_angle             = 30[deg]
X_atn               = 8[dB]
X_pulse             = 3.73333333[us]
Irr_atn_dec         = 21.2[dB]
Irr_atn_noe         = 21.2[dB]
Irr_noise           = WALTZ
Decoupling          = TRUE
Initial_wait        = 1[s]
Noe                 = TRUE
Noe_time            = 2[s]
Recvr_gain          = 60
Relaxation_delay    = 2[s]
Repetition_time     = 2.69206016[s]
Temp_get            = -80[dC]
```

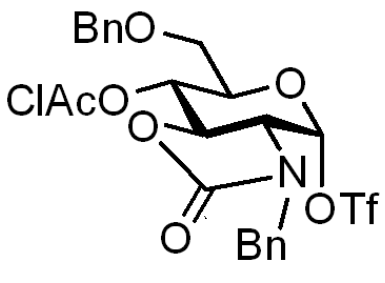

2b

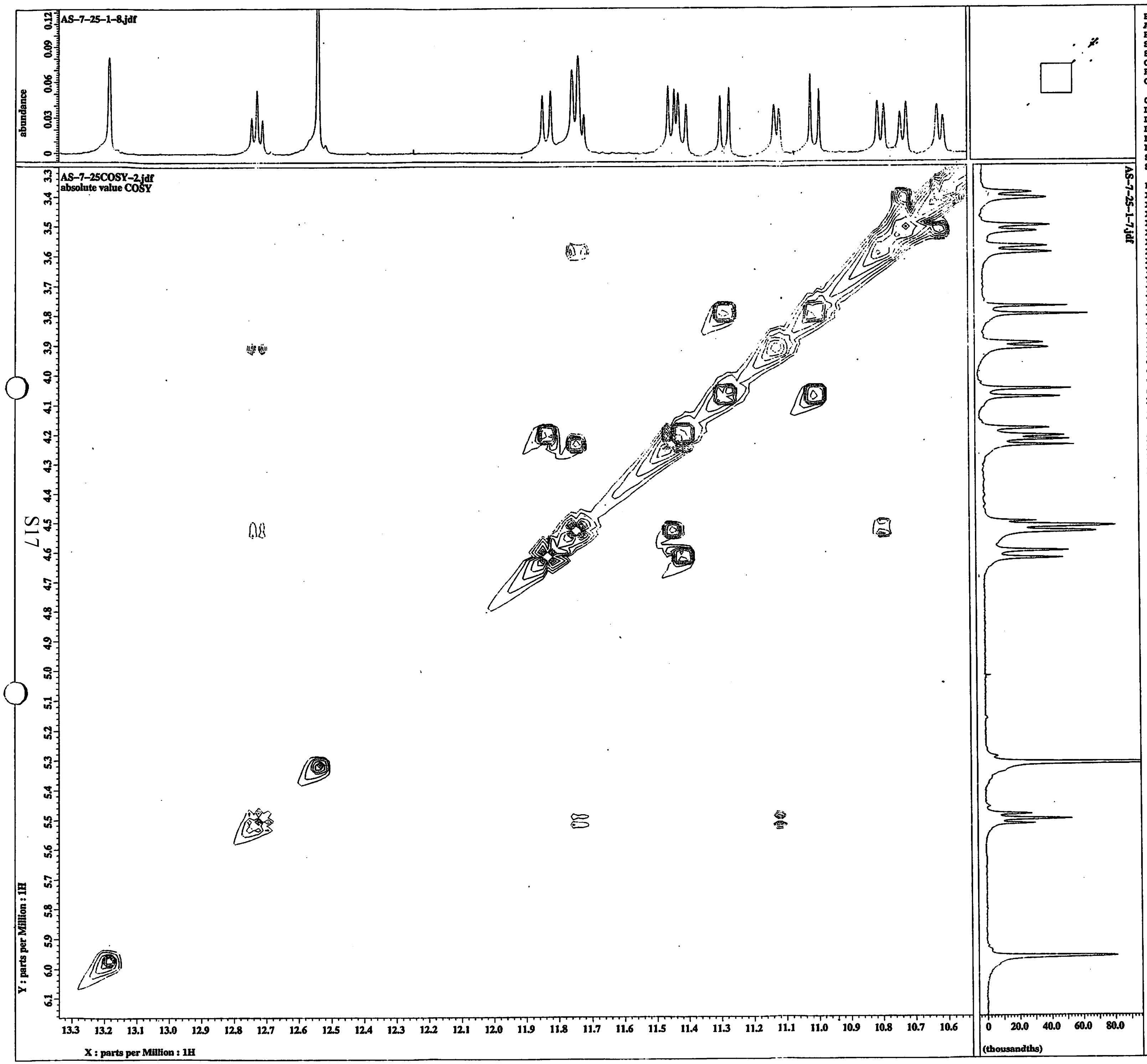

Filename	= AS-7-25COSY-2.jdf
Author	= delta
Experiment	= cosy.ex2
Sample_id	= S#483381
Solvent	= METHYLENE-CHLORI
Creation_time	= 26-SEP-2007 13:54:12
Revision_time	= 26-SEP-2007 13:57:26
Current_time	= 26-SEP-2007 13:59:14
Content	= absolute value COSY
Data_format	= 2D REAL REAL
Dim_size	= 1024, 1024
Dim_title	= 1H 1H
Dim_units	= [ppm] [ppm]
Dimensions	= X Y
Site	= ECA600
Spectrometer	= JNM-ECA600
Field_strength	= 14.09636928[T] (600[M
X_acq_duration	= 0.113664[s]
X_domain	= 1H
X_freq	= 600.1723046[MHz]
X_offset	= 5[ppm]
X_points	= 1280
X_prescans	= 4
X_resolution	= 8.79786036[Hz]
X_sweep	= 11.26126126[kHz]
Y_domain	= 1H
Y_freq	= 600.1723046[MHz]
Y_offset	= 5[ppm]
Y_points	= 256
Y_prescans	= 0
Y_resolution	= 35.17242932[Hz]
Y_sweep	= 9.00414191[kHz]
Irr_domain	= 1H
Irr_freq	= 600.1723046[MHz]
Irr_offset	= 5[ppm]
Tri_domain	= 1H
Tri_freq	= 600.1723046[MHz]
Tri_offset	= 5[ppm]
Clipped	= TRUE
Mod_return	= 1
Scans	= 4
Total_scans	= 1024
X_90_width	= 13.5[us]
X_acq_time	= 0.113664[s]
X_atn	= 7.8[dB]
X_pulse	= 13.5[us]
Y_acq_time	= 28.43136[ms]
Irr_mode	= Off
Tri_mode	= Off
Dante_presat	= FALSE
Delta	= 0[ms]
Initial_wait	= 1[s]
Pulse_1	= 13.5[us]
Pulse_2	= 13.5[us]
Pulse_angle_1	= 90[deg]
Pulse_angle_2	= 90[deg]
Recvr_gain	= 48
Relaxation_delay	= 1.5[s]
Repetition_time	= 1.613664[s]
T1	= 1[us]
Temp_get	= -80[dC]

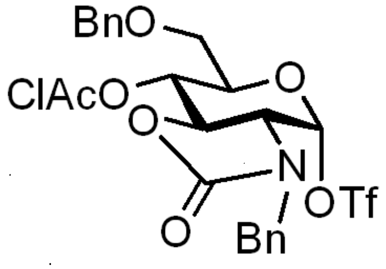

2b

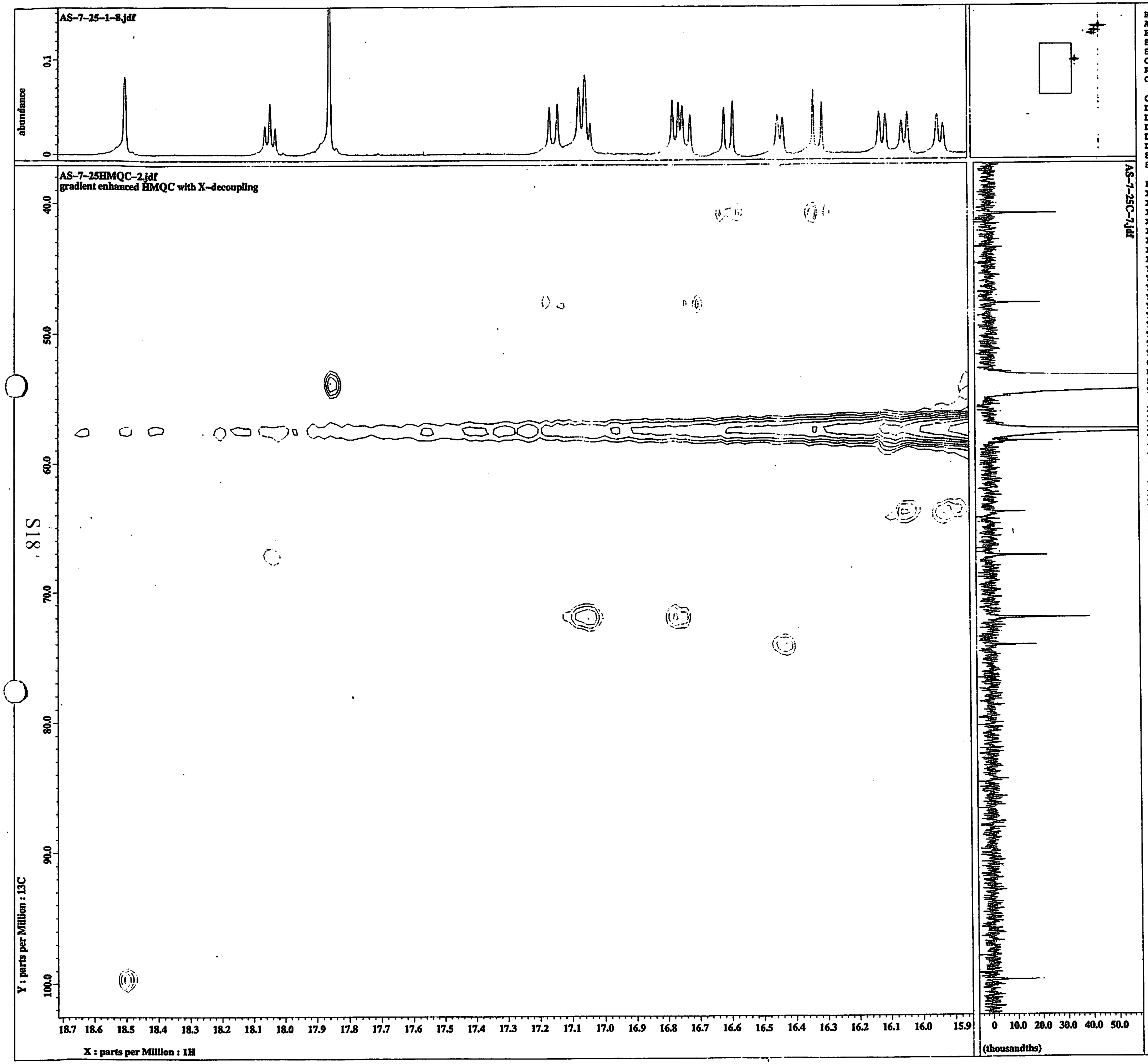

Filename	= AS-7-25HMQC-2.jdf
Author	= delta
Experiment	= hmqc_pfg.ex2
Sample_id	= S#675452
Solvent	= METHYLENE-CHLORI
Creation_time	= 26-SEP-2007 19:13:52
Revision_time	= 26-SEP-2007 19:17:11
Current_time	= 26-SEP-2007 19:19:06
Content	= gradient enhanced HMQ
Data_format	= 2D REAL REAL
Dim_size	= 819, 512
Dim_title	= 1H 13C
Dim_units	= [ppm] [ppm]
Dimensions	= X Y
Site	= ECA600
Spectrometer	= JNM-ECA600
Field_strength	= 14.09636928[T] (600[M
X_acq_duration	= 90.9312[ms]
X_domain	= 1H
X_freq	= 600.1723046[MHz]
X_offset	= 5[ppm]
X_points	= 1024
X_prescans	= 4
X_resolution	= 10.99732545[Hz]
X_sweep	= 11.26126126[kHz]
Y_domain	= 13C
Y_freq	= 150.91343039[MHz]
Y_offset	= 85[ppm]
Y_points	= 256
Y_prescans	= 0
Y_resolution	= 117.94233092[Hz]
Y_sweep	= 30.19323671[kHz]
Tri_domain	= 1H
Tri_freq	= 600.1723046[MHz]
Tri_offset	= 5[ppm]
Clipped	= FALSE
Mod_return	= 1
Scans	= 4
Total_scans	= 1024
X_acq_time	= 90.9312[ms]
X_atn	= 7.8[dB]
X_pulse	= 13.5[us]
Y_acq_time	= 8.47872[ms]
Y_atn	= 8[dB]
Y_pulse	= 11.2[us]
Irr_atn_dec	= 24.4[dB]
Irr_noise	= MPF8
Tri_mode	= Off
Dante_presat	= FALSE
Decoupling	= TRUE
Grad_1	= 1[ms]
Grad_1_amp	= 60[%]
Grad_2	= 1[ms]
Grad_2_amp	= 60[%]
Grad_3	= 1[ms]
Grad_3_amp	= 30.18108652[%]
Grad_recover	= 0.1[ms]
Grad_selection	= 13C = 1.988:1.98
Grad_shape	= SINE
Initial_wait	= 1[s]
J_constant	= 140[Hz]
Recvr_gain	= 84
Refocus_comp	= 2.57142857[ms]
Relaxation_delay	= 1.5[s]
Repetition_time	= 1.5909312[s]
T1	= 1[us]
Temp1	= 1.988
Temp_get	= -80[dC]

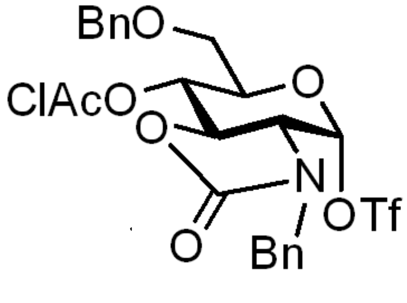

2b

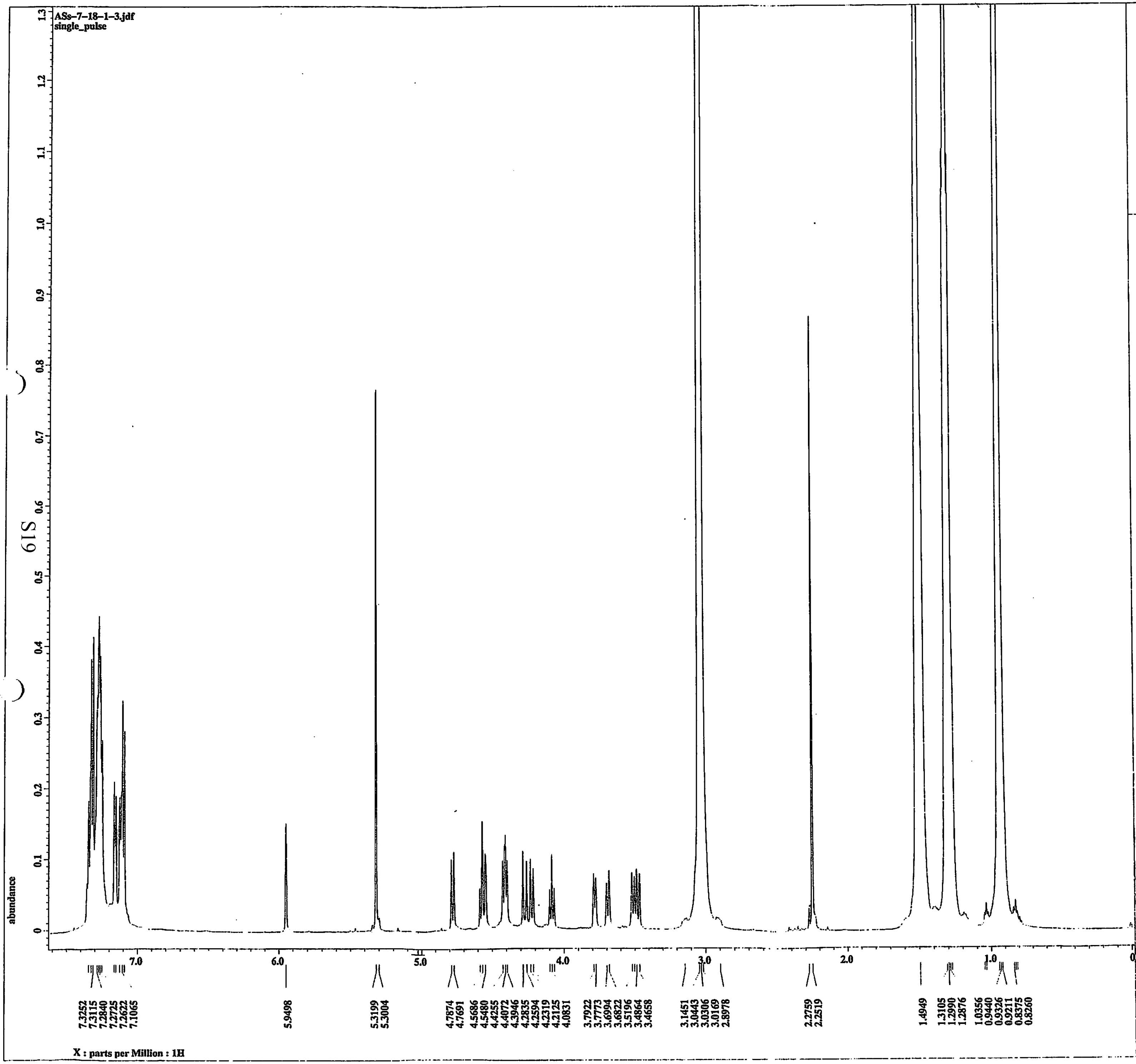


```
---- PROCESSING PARAMETERS ----
dc_balance : 0 : FALSE
sexp : 0.2[Hz] : 0.0[s]
trapezoid3 : 0[%] : 80[%] : 100[%]
zerofill : 1
fft : 1 : TRUE : TRUE
machinephase
ppm

Derived from: ASs-7-18-1-1.jdf

Filename           = ASs-7-18-[illegible].jdf
Author             = delta
Experiment         = single_pulse.ex2
Sample_id          = S#482268
Solvent            = METHYLENE-CHLORI
Creation_time      = 14-SEP-2007 13:29:26
Revision_time      = 14-SEP-2007 13:30:32
Current_time       = 14-SEP-2007 13:30:43

Content            = single_pulse
Data_format        = 1D COMPLEX
Dim_size           = 13107
Dim_title          = 1H
Dim_units          = [ppm]
Dimensions         = X
Site               = ECA600
Spectrometer       = JNM-ECA600

Field_strength     = 14.09636928[T] (600[M
X_acq_duration     = 1.4548992[s]
X_domain           = 1H
X_freq             = 600.1723046[MHz]
X_offset           = 5[ppm]
X_points           = 16384
X_prescans         = 1
X_resolution       = 0.68733284[Hz]
X_sweep            = 11.26126126[kHz]
Irr_domain         = 1H
Irr_freq           = 600.1723046[MHz]
Irr_offset         = 5[ppm]
Tri_domain         = 1H
Tri_freq           = 600.1723046[MHz]
Tri_offset         = 5[ppm]
Clipped            = FALSE
Mod_return         = 1
Scans              = 32
Total_scans        = 32

X_90_width         = 13.5[us]
X_acq_time         = 1.4548992[s]
X_angle            = 45[deg]
X_atn              = 7.8[dB]
X_pulse            = 6.75[us]
Irr_mode           = Off
Tri_mode           = Off
Dante_presat       = FALSE
Initial_wait       = 1[s]
Recvr_gain         = 40
Relaxation_delay   = 5[s]
Repetition_time    = 6.4548992[s]
Temp_get           = -80[dC]
```

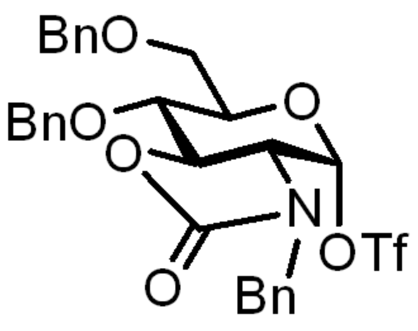

2c

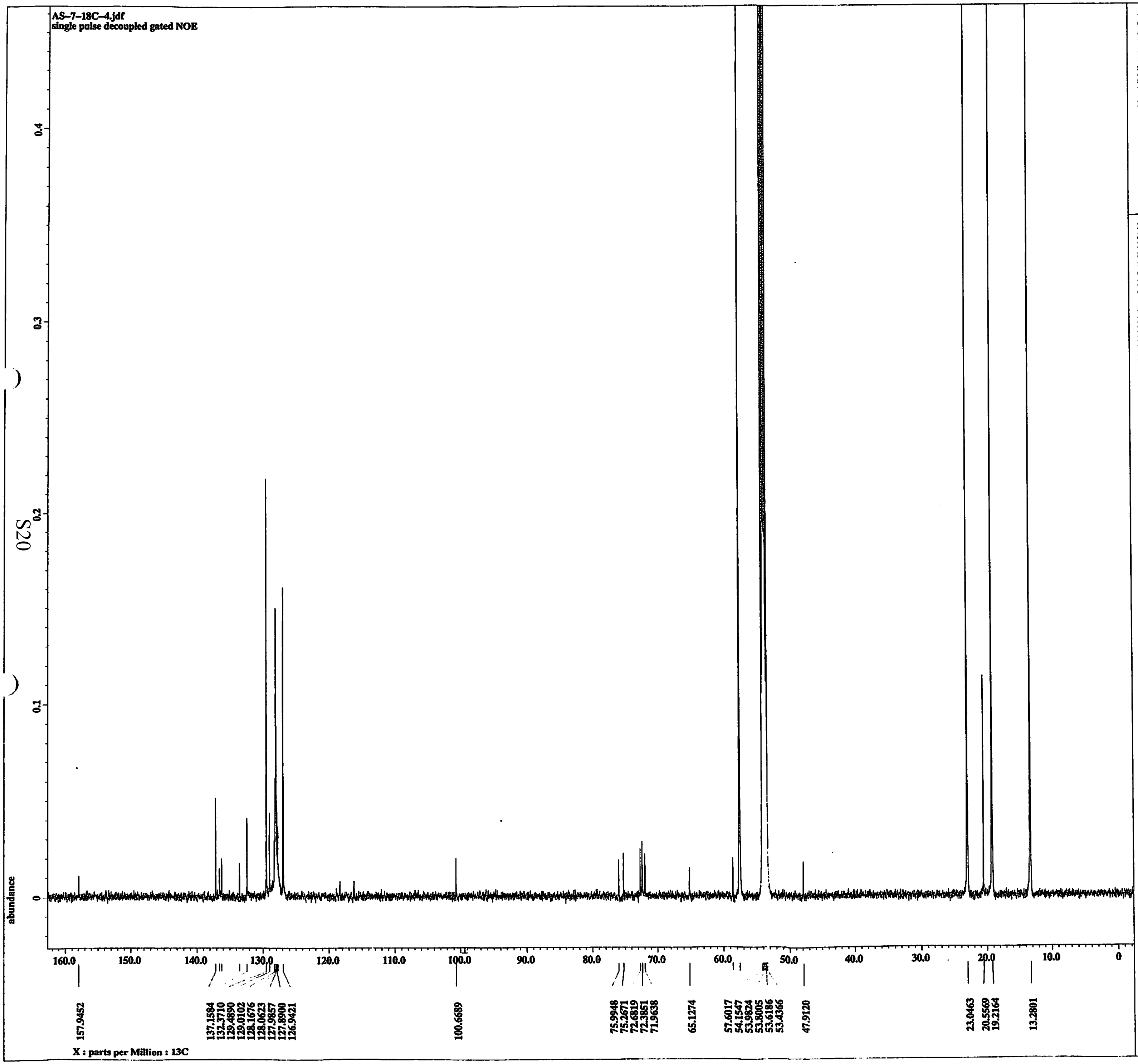


```
---- PROCESSING PARAMETERS ----
dc_balance : 0 : FALSE
sexp : 2.0[Hz] : 0.0[s]
trapezoid3 : 0[%] : 80[%] : 100[%]
zerofill : 1
fft : 1 : TRUE : TRUE
machinephase
ppm
base_correct : None : 0 : Smooth

Derived from: AS-7-18C-1.jdf
```

```
Filename          = AS-7-18C-4.jdf
Author            = delta
Experiment        = single_pulse_dec
Sample_id         = S0489003
Solvent           = METHYLENE-CHLORI
Creation_time     = 14-SEP-2007 17:17:58
Revision_time     = 14-SEP-2007 17:26:54
Current_time      = 14-SEP-2007 17:27:13

Content           = single pulse decouple
Data_format       = 1D REAL
Dim_size          = 26214
Dim_title         = 13C
Dim_units         = [ppm]
Dimensions        = X
Site              = ECA600
Spectrometer      = JNM-ECA600

Field_strength    = 14.09636928[T] (600[M
X_acq_duration    = 0.69206016[s]
X_domain          = 13C
X_freq            = 150.91343039[MHz]
X_offset          = 100[ppm]
X_points          = 32768
X_prescans        = 4
X_resolution      = 1.44496109[Hz]
X_sweep           = 47.34848485[kHz]
Irr_domain        = 1H
Irr_freq          = 600.1723046[MHz]
Irr_offset        = 5[ppm]
Clipped           = FALSE
Mod_return        = 1
Scans             = 4915
Total_scans       = 4915

X_90_width        = 11.2[us]
X_acq_time        = 0.69206016[s]
X_angle           = 30[deg]
X_atn             = 8[dB]
X_pulse           = 3.73333333[us]
Irr_atn_dec       = 21.2[dB]
Irr_atn_noe       = 21.2[dB]
Irr_noise         = WALTZ
Decoupling        = TRUE
Initial_wait      = 1[s]
Noe               = TRUE
Noe_time          = 2[s]
Recvr_gain        = 60
Relaxation_delay  = 2[s]
Repetition_time   = 2.69206016[s]
Temp_get          = -80[dC]
```

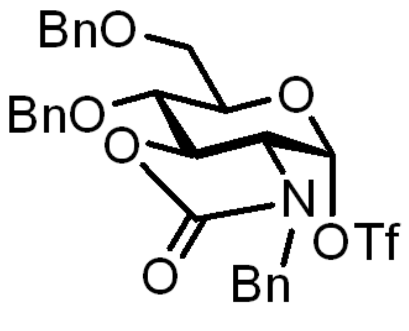

2c

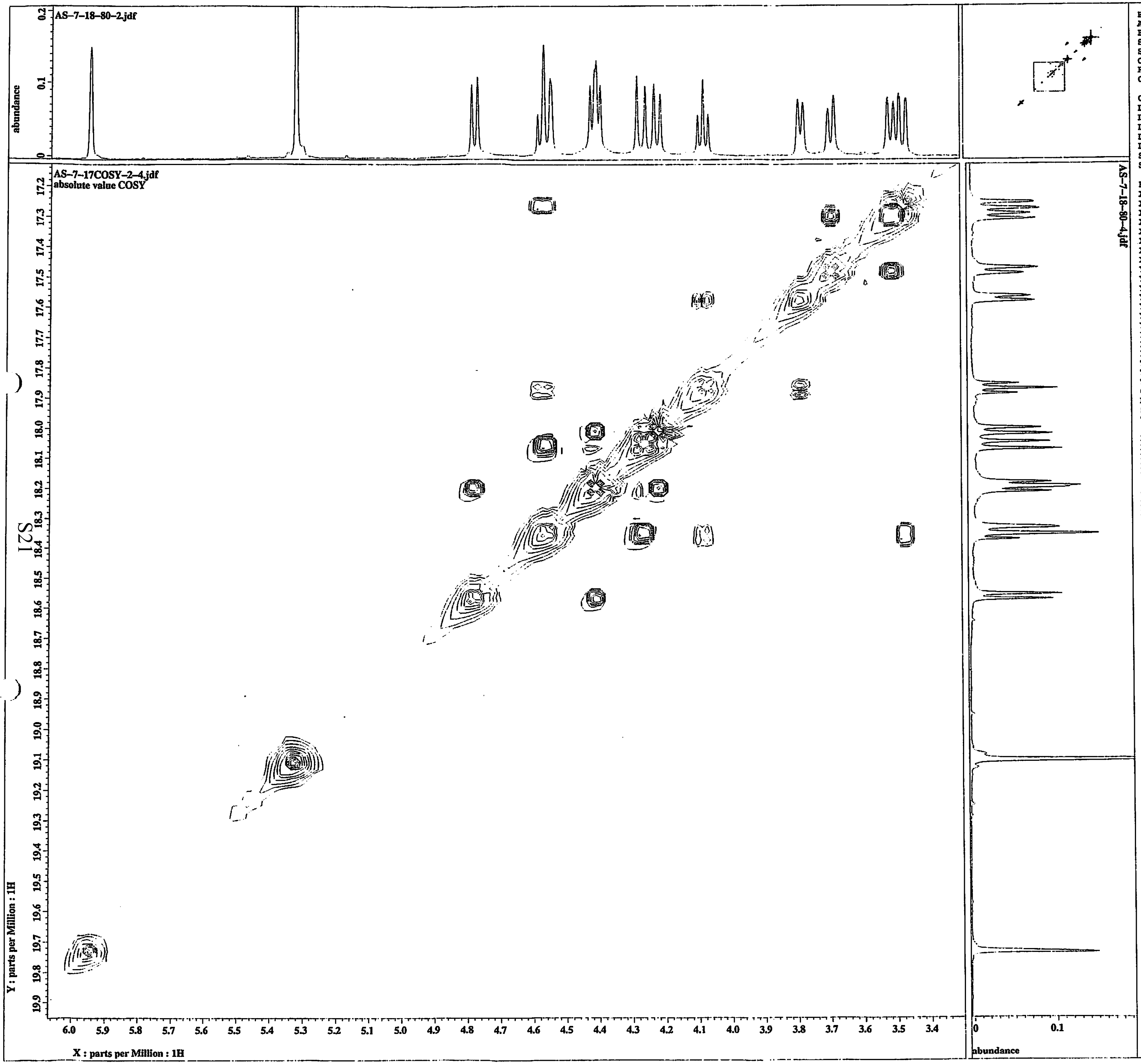

Filename	= AS-7-17COSY-2-4.jdf
Author	= delta
Experiment	= cosy.ex2
Sample_id	= S#776007
Solvent	= METHYLENE-CHLORI
Creation_time	= 13-SEP-2007 22:02:39
Revision_time	= 14-SEP-2007 17:14:31
Current_time	= 14-SEP-2007 17:15:31
Content	= absolute value COSY
Data_format	= 2D REAL REAL
Dim_size	= 1024, 1024
Dim_title	= 1H 1H
Dim_units	= [ppm] [ppm]
Dimensions	= X Y
Site	= ECA600
Spectrometer	= JNM-ECA600
Field_strength	= 14.09636928[T] (600[M
X_acq_duration	= 0.113664[s]
X_domain	= 1H
X_freq	= 600.1723046[MHz]
X_offset	= 5[ppm]
X_points	= 1280
X_prescans	= 4
X_resolution	= 8.79786036[Hz]
X_sweep	= 11.26126126[kHz]
Y_domain	= 1H
Y_freq	= 600.1723046[MHz]
Y_offset	= 5[ppm]
Y_points	= 256
Y_prescans	= 0
Y_resolution	= 35.17242932[Hz]
Y_sweep	= 9.00414191[kHz]
Irr_domain	= 1H
Irr_freq	= 600.1723046[MHz]
Irr_offset	= 5[ppm]
Tri_domain	= 1H
Tri_freq	= 600.1723046[MHz]
Tri_offset	= 5[ppm]
Clipped	= TRUE
Mod_return	= 1
Scans	= 4
Total_scans	= 1024
X_90_width	= 13.5[us]
X_acq_time	= 0.113664[s]
X_atn	= 7.8[dB]
X_pulse	= 13.5[us]
Y_acq_time	= 28.43136[ms]
Irr_mode	= Off
Tri_mode	= Off
Dante_presat	= FALSE
Delta	= 0[ms]
Initial_wait	= 1[s]
Pulse_1	= 13.5[us]
Pulse_2	= 13.5[us]
Pulse_angle_1	= 90[deg]
Pulse_angle_2	= 90[deg]
Recvr_gain	= 50
Relaxation_delay	= 1.5[s]
Repetition_time	= 1.613664[s]
T1	= 1[us]
Temp_get	= -80[dC]

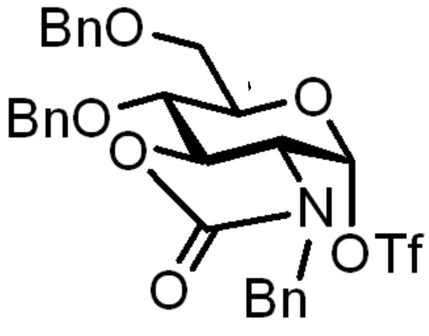

2c

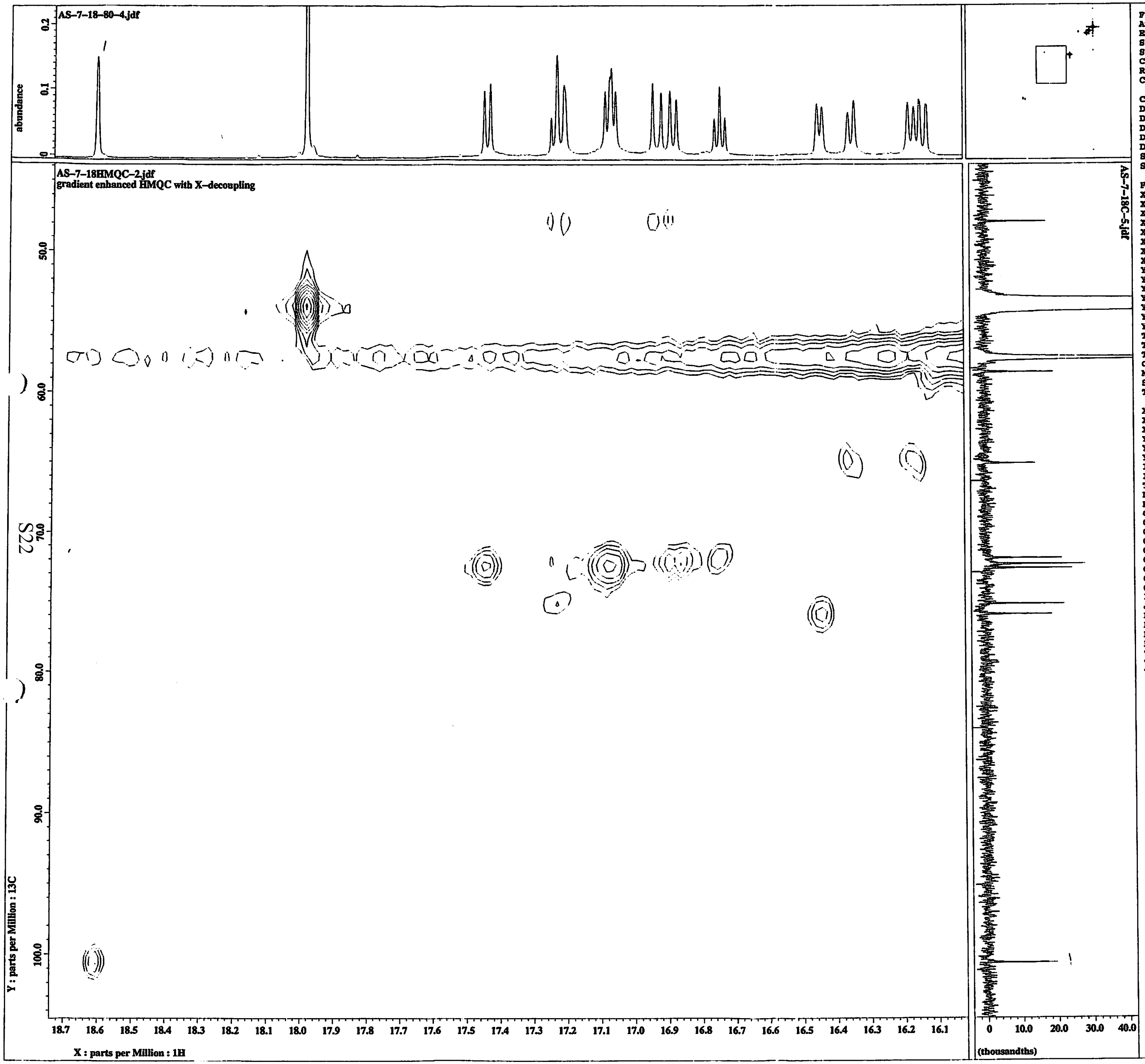

Filename	= AS-7-18HMQC-2.jdf
Author	= delta
Experiment	= hmqc_pfg.ex2
Sample_id	= S8629913
Solvent	= METHYLENE-CHLORI
Creation_time	= 14-SEP-2007 17:59:28
Revision_time	= 14-SEP-2007 18:02:21
Current_time	= 14-SEP-2007 18:05:37
Content	= gradient enhanced HMQ
Data_format	= 2D REAL REAL
Dim_size	= 819, 512
Dim_title	= 1H 13C
Dim_units	= [ppm] [ppm]
Dimensions	= X Y
Site	= ECA600
Spectrometer	= JNM-ECA600
Field_strength	= 14.09636928[T] (600[M
X_acq_duration	= 90.9312[ms]
X_domain	= 1H
X_freq	= 600.1723046[MHz]
X_offset	= 5[ppm]
X_points	= 1024
X_prescans	= 4
X_resolution	= 10.99732545[Hz]
X_sweep	= 11.26126126[kHz]
Y_domain	= 13C
Y_freq	= 150.91343039[MHz]
Y_offset	= 100[ppm]
Y_points	= 256
Y_prescans	= 0
Y_resolution	= 147.51699396[Hz]
Y_sweep	= 37.76435045[kHz]
Tri_domain	= 1H
Tri_freq	= 600.1723046[MHz]
Tri_offset	= 5[ppm]
Clipped	= TRUE
Mod_return	= 1
Scans	= 4
Total_scans	= 1024
X_acq_time	= 90.9312[ms]
X_atn	= 7.8[dB]
X_pulse	= 13.5[us]
Y_acq_time	= 6.77888[ms]
Y_atn	= 8[dB]
Y_pulse	= 11.2[us]
Irr_atn_dec	= 24.4[dB]
Irr_noise	= MPF8
Tri_mode	= Off
Dante_presat	= FALSE
Decoupling	= TRUE
Grad_1	= 1[ms]
Grad_1_amp	= 60[%]
Grad_2	= 1[ms]
Grad_2_amp	= 60[%]
Grad_3	= 1[ms]
Grad_3_amp	= 30.18108652[%]
Grad_recover	= 0.1[ms]
Grad_selection	= 13C = 1.988:1.98
Grad_shape	= SINE
Initial_wait	= 1[s]
J_constant	= 140[Hz]
Recvr_gain	= 86
Refocus_comp	= 2.57142857[ms]
Relaxation_delay	= 1.5[s]
Repetition_time	= 1.5909312[s]
T1	= 1[us]
Temp1	= 1.988
Temp_get	= -80[dC]

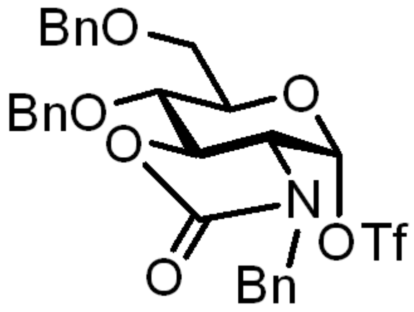

2c

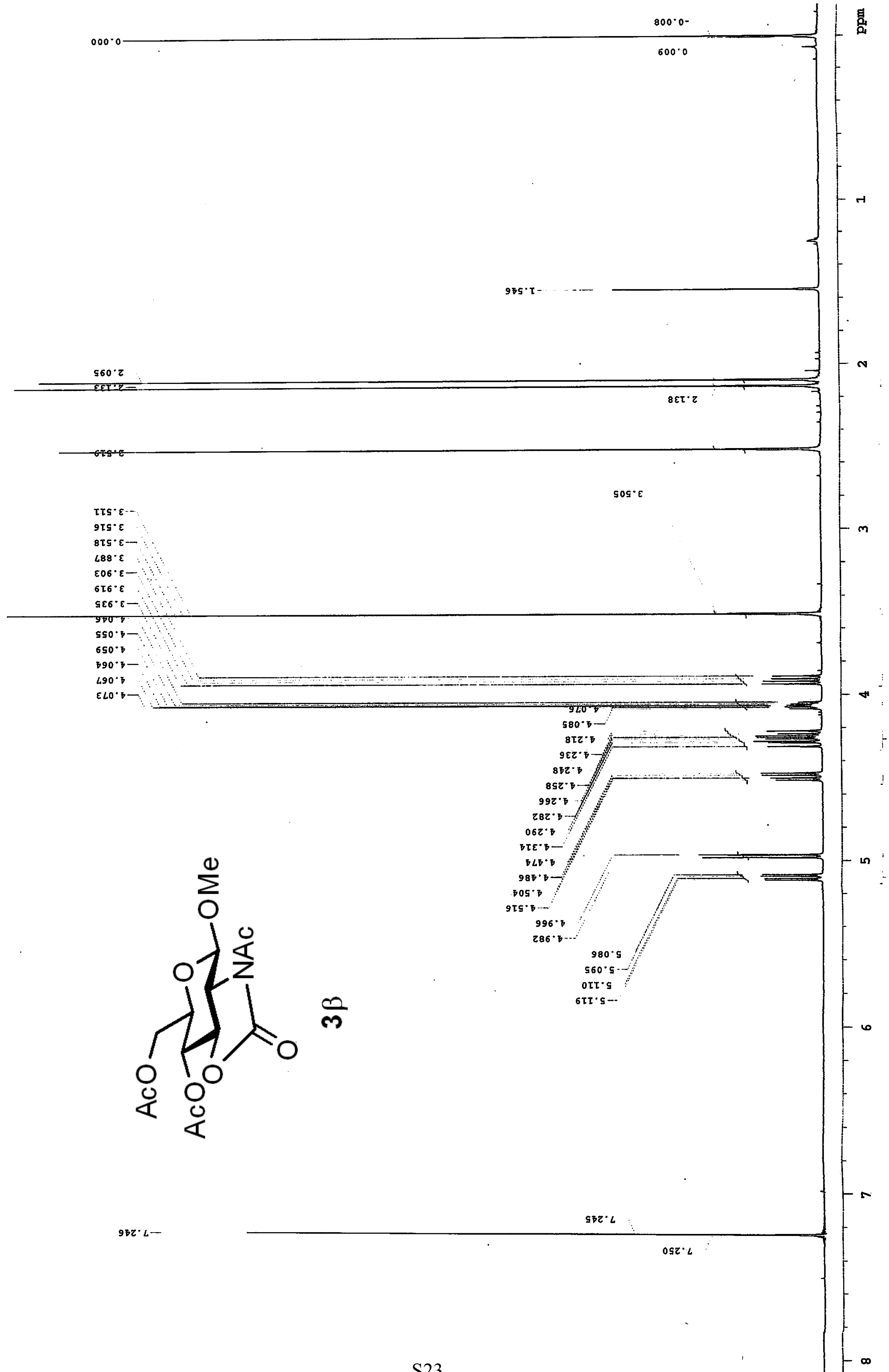
OMe
NAc
AcO
AcO
3β
ppm

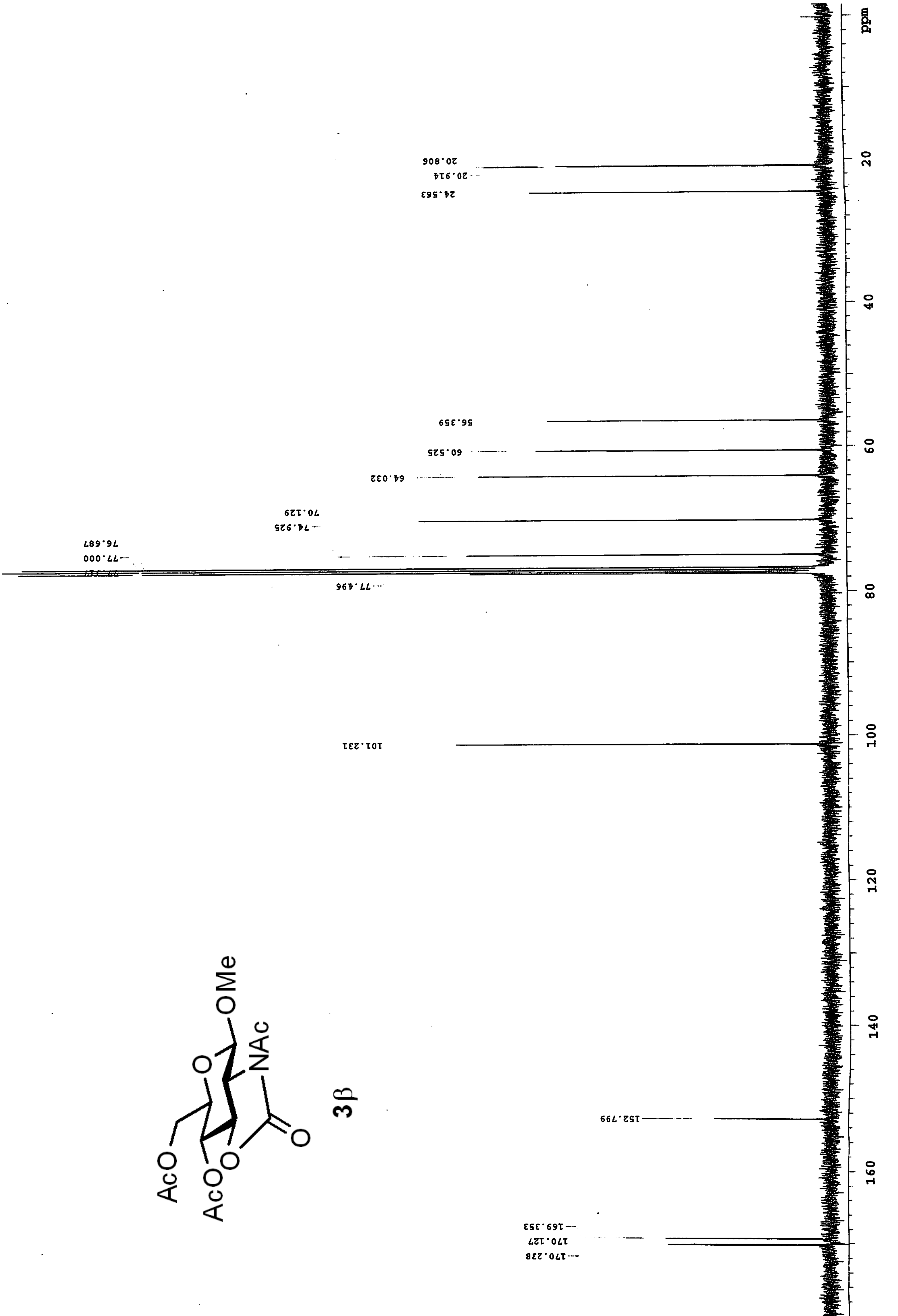
20.806
20.914
24.563
56.359
60.525
64.032
70.129
74.925
76.687
77.000
77.496
101.231
152.799
169.353
170.127
170.238
ppm
20
40
60
80
100
120
140
160
OMe
NAc
O
AcO
AcO
3β

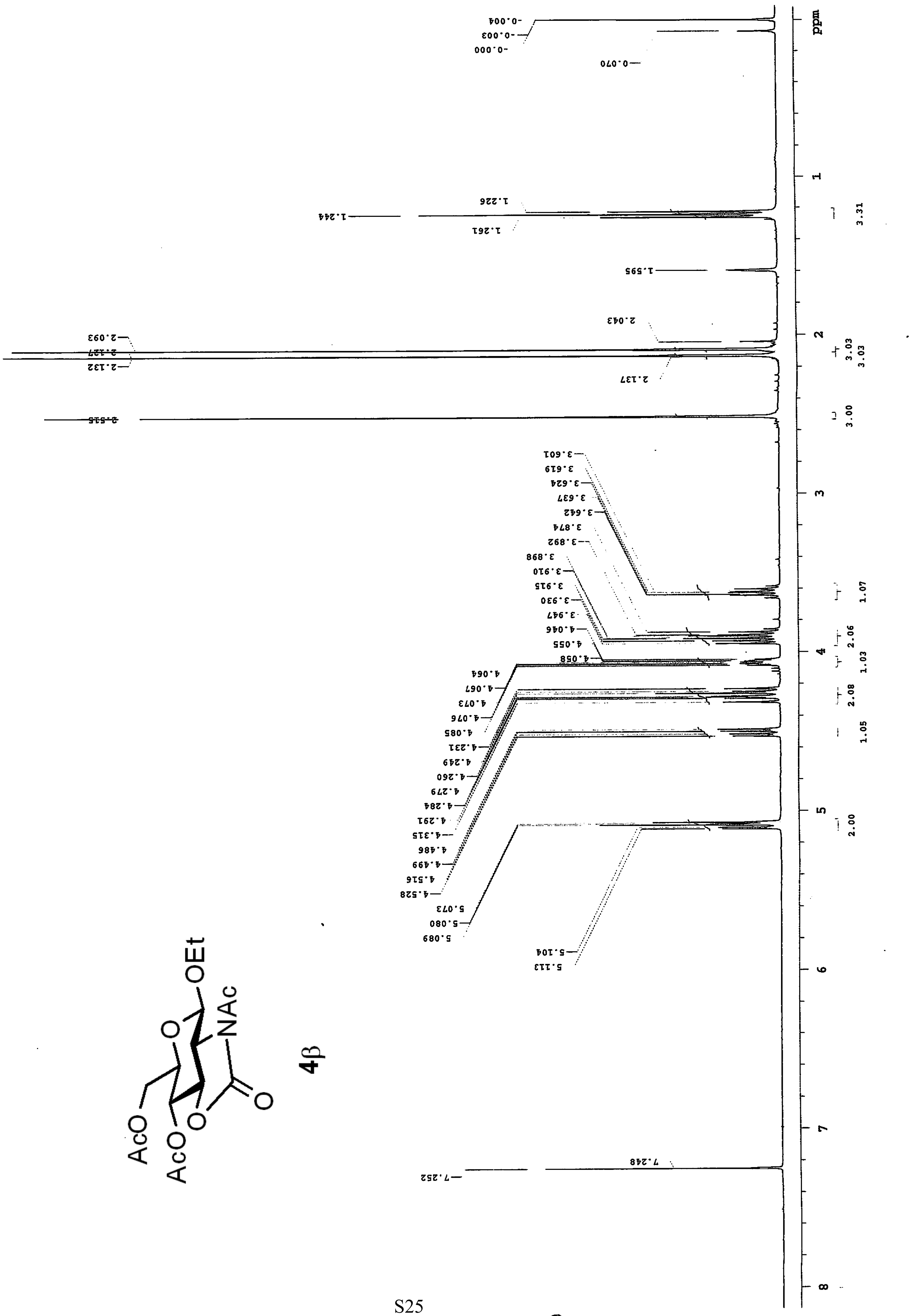
ppm
-0.004
-0.003
-0.000
0.070
1.226
1.244
1.261
1.595
2.043
2.093
2.127
2.132
2.137
2.515
3.601
3.619
3.624
3.637
3.642
3.874
3.892
3.898
3.910
3.915
3.930
3.947
4.046
4.055
4.058
4.064
4.067
4.073
4.076
4.085
4.231
4.249
4.260
4.279
4.284
4.291
4.315
4.486
4.499
4.516
4.528
5.073
5.080
5.089
5.104
5.113
7.248
7.252
3.31
3.03
3.03
3.00
1.07
2.06
1.03
2.08
1.05
2.00
AcO
AcO
OEt
NAc
O

4β

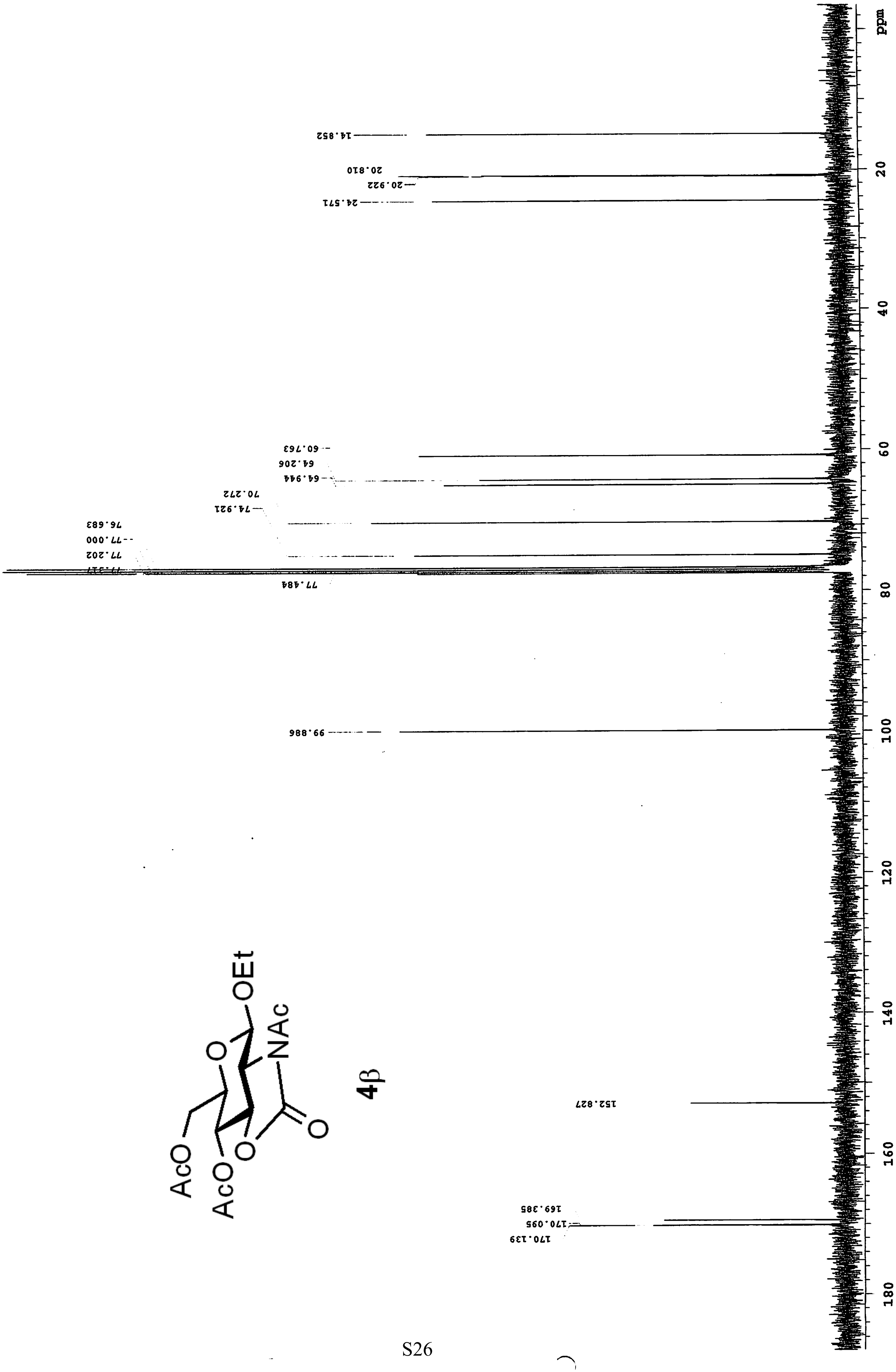

14.852
20.810
20.922
24.571
60.763
64.206
64.944
70.272
74.921
76.683
77.000
77.202
77.317
77.484
99.886
152.827
169.385
170.095
170.139
ppm
20
40
60
80
100
120
140
160
180
AcO
AcO
O
O
O
OEt
NAc
O
4β

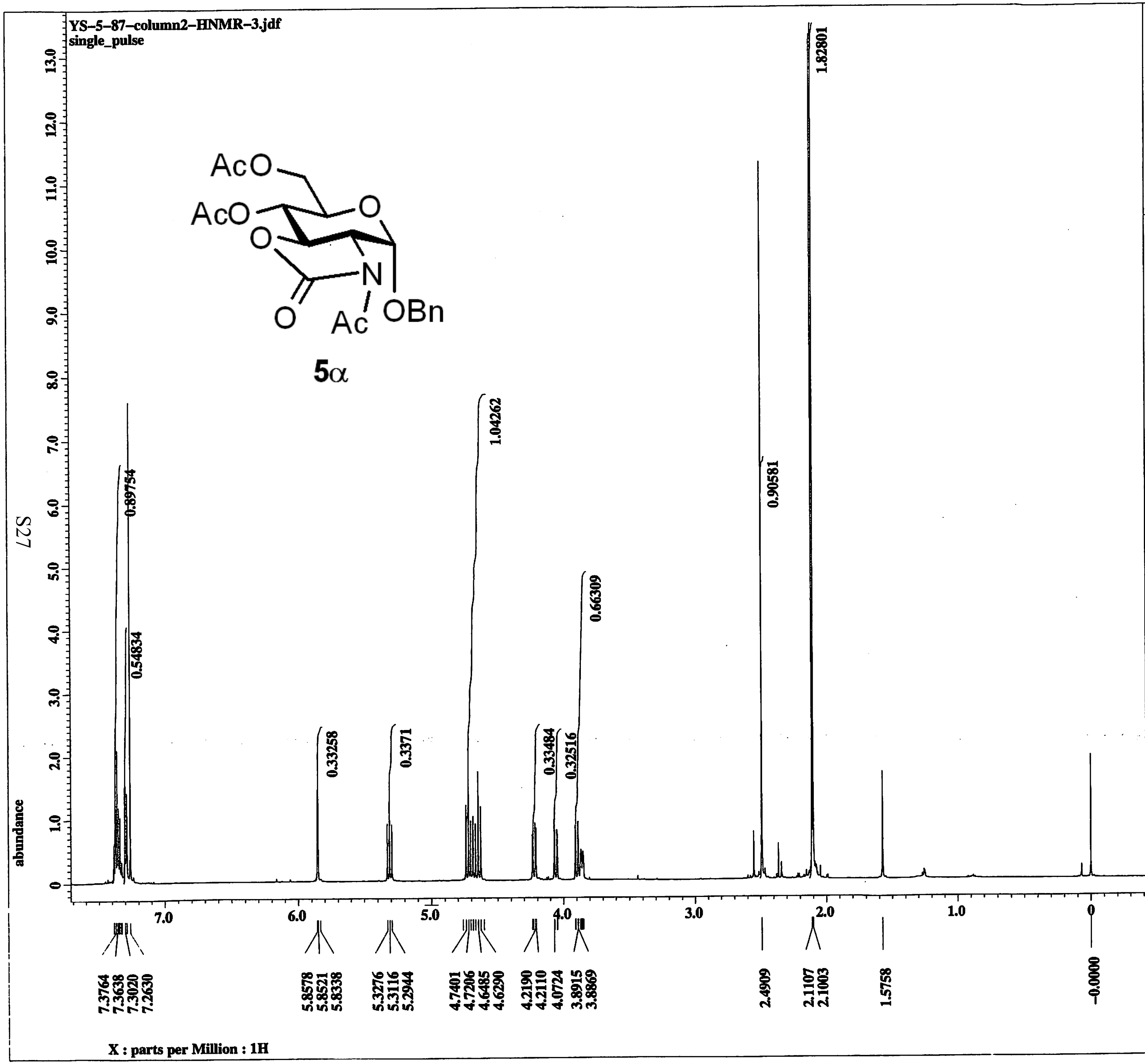


```
---- PROCESSING PARAMETERS ----
dc_balance : 0 : FALSE
sexp : 0.2[Hz] : 0.0[s]
trapezoid3 : 0[%] : 80[%] : 100[%]
zerofill : 1
fft : 1 : TRUE : TRUE
machinephase
ppm

Derived from: YS-5-87-column2-HNMR-1.jdf
```

```
Filename          = YS-5-87-column2-HNMR-
Author            = delta
Experiment        = single_pulse.ex2
Sample_id         = YS-5-87-column2
Solvent           = CHLOROFORM-D
Creation_time     = 14-DEC-2011 23:11:20
Revision_time     = 20-DEC-2011 21:30:40
Current_time      = 20-DEC-2011 21:30:48

Content           = single_pulse
Data_format       = 1D COMPLEX
Dim_size          = 13107
Dim_title         = 1H
Dim_units         = [ppm]
Dimensions        = X
Site              = ECA600
Spectrometer      = JNM-ECA600

Field_strength    = 14.09636928[T] (600[M
X_acq_duration    = 1.4548992[s]
X_domain          = 1H
X_freq            = 600.1723046[MHz]
X_offset          = 5[ppm]
X_points          = 16384
X_prescans        = 1
X_resolution      = 0.68733284[Hz]
X_sweep           = 11.26126126[kHz]
Irr_domain        = 1H
Irr_freq          = 600.1723046[MHz]
Irr_offset        = 5[ppm]
Tri_domain        = 1H
Tri_freq          = 600.1723046[MHz]
Tri_offset        = 5[ppm]
Clipped           = FALSE
Mod_return        = 1
Scans             = 16
Total_scans       = 16

X_90_width        = 11.2[us]
X_acq_time        = 1.4548992[s]
X_angle           = 45[deg]
X_atn             = 4.2[dB]
X_pulse           = 5.6[us]
Irr_mode          = Off
Tri_mode          = Off
Dante_presat      = FALSE
Initial_wait      = 1[s]
Recvr_gain        = 52
Relaxation_delay  = 5[s]
Repetition_time   = 6.4548992[s]
Temp_get          = 21.2[dC]
```

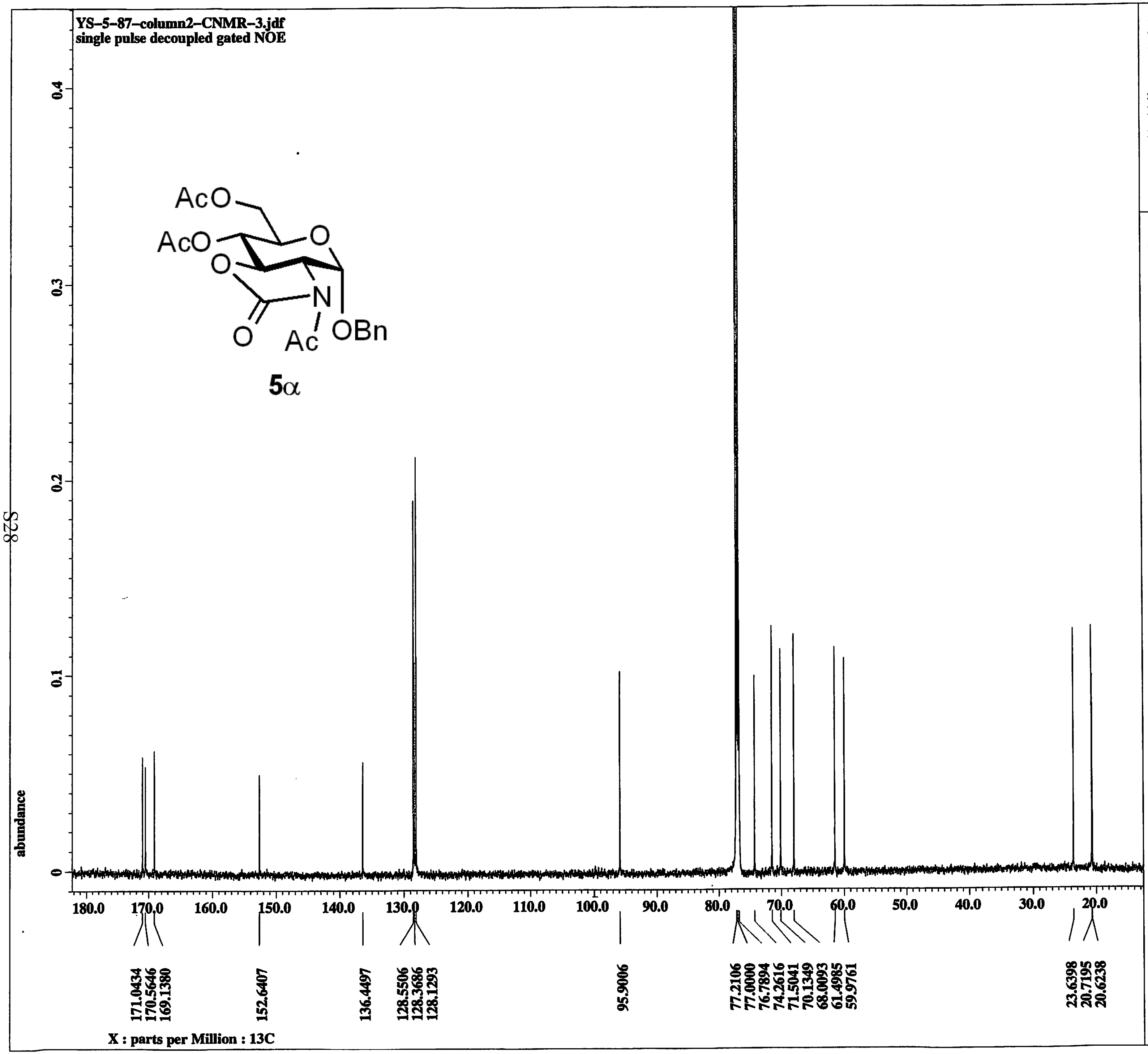


```
---- PROCESSING PARAMETERS ----
dc_balance : 0 : FALSE
sexp : 2.0[Hz] : 0.0[s]
trapezoid3 : 0[%] : 80[%] : 100[%]
zerofill : 1
fft : 1 : TRUE : TRUE
machinephase
ppm

Derived from: YS-5-87-column2-CNMR-1.jdf
```

```
Filename            = YS-5-87-column2-CNMR-
Author              = delta
Experiment          = single_pulse_dec
Sample_id           = YS-5-87-column2
Solvent             = CHLOROFORM-D
Creation_time       = 15-DEC-2011 06:42:02
Revision_time       = 15-DEC-2011 12:57:07
Current_time        = 15-DEC-2011 12:57:27

Content             = single pulse decouple
Data_format         = 1D COMPLEX
Dim_size            = 26214
Dim_title           = 13C
Dim_units           = [ppm]
Dimensions          = X
Site                = ECA600
Spectrometer        = JNM-ECA600

Field_strength      = 14.09636928[T] (600[M
X_acq_duration      = 0.69206016[s]
X_domain            = 13C
X_freq              = 150.91343039[MHz]
X_offset            = 100[ppm]
X_points            = 32768
X_prescans          = 4
X_resolution        = 1.44496109[Hz]
X_sweep             = 47.34848485[kHz]
Irr_domain          = 1H
Irr_freq            = 600.1723046[MHz]
Irr_offset          = 5[ppm]
Clipped             = TRUE
Mod_return          = 1
Scans               = 10000
Total_scans         = 10000

X_90_width          = 10.5[us]
X_acq_time          = 0.69206016[s]
X_angle             = 30[deg]
X_atn               = 8[dB]
X_pulse             = 3.5[us]
Irr_atn_dec         = 21.494[dB]
Irr_atn_noe         = 21.494[dB]
Irr_noise           = WALTZ
Decoupling          = TRUE
Initial_wait        = 1[s]
Noe                 = TRUE
Noe_time            = 2[s]
Recvr_gain          = 60
Relaxation_delay    = 2[s]
Repetition_time     = 2.69206016[s]
Temp_get            = 22[dC]
```

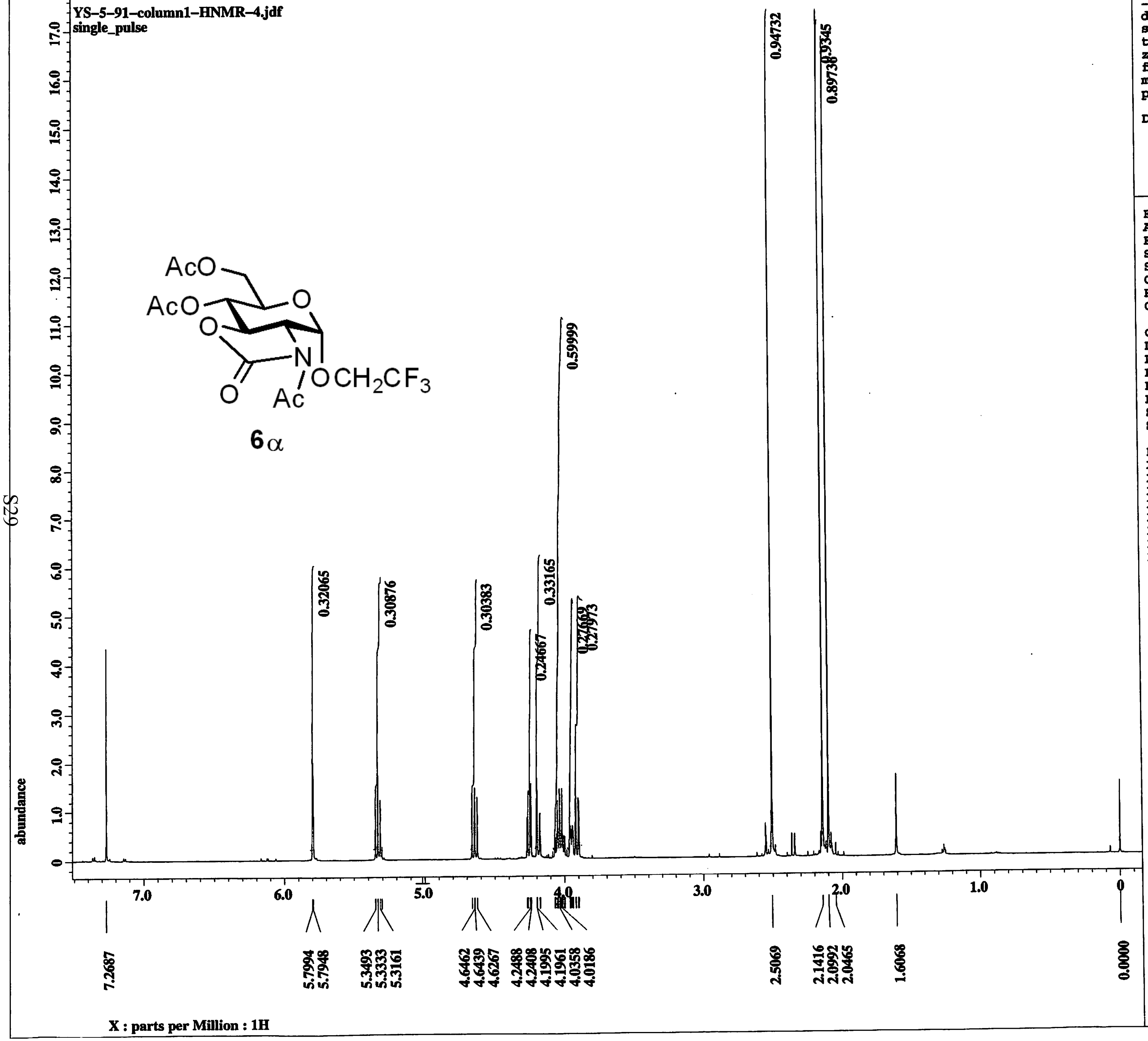


```
---- PROCESSING PARAMETERS ----
dc_balance : 0 : FALSE
sexp : 0.2[Hz] : 0.0[s]
trapezoid3 : 0[%] : 80[%] : 100[%]
zerofill : 1
fft : 1 : TRUE : TRUE
machinephase
ppm

Derived from: YS-5-91-column1-HNMR-1.jdf

Filename            = YS-5-91-column1-HNMR-
Author              = delta
Experiment          = single_pulse.ex2
Sample_id           = YS-5-91-column1
Solvent             = CHLOROFORM-D
Creation_time       = 20-DEC-2011 21:11:12
Revision_time       = 20-DEC-2011 21:33:41
Current_time        = 20-DEC-2011 21:34:09

Content             = single_pulse
Data_format         = 1D COMPLEX
Dim_size            = 13107
Dim_title           = 1H
Dim_units           = [ppm]
Dimensions          = X
Site                = ECA600
Spectrometer        = JNM-ECA600

Field_strength      = 14.09636928[T] (600[M
X_acq_duration      = 1.4548992[s]
X_domain            = 1H
X_freq              = 600.1723046[MHz]
X_offset            = 5[ppm]
X_points            = 16384
X_prescans          = 1
X_resolution        = 0.68733284[Hz]
X_sweep             = 11.26126126[kHz]
Irr_domain          = 1H
Irr_freq            = 600.1723046[MHz]
Irr_offset          = 5[ppm]
Tri_domain          = 1H
Tri_freq            = 600.1723046[MHz]
Tri_offset          = 5[ppm]
Clipped             = FALSE
Mod_return          = 1
Scans               = 16
Total_scans         = 16

X_90_width          = 11.2[us]
X_acq_time          = 1.4548992[s]
X_angle             = 45[deg]
X_atn               = 4.2[dB]
X_pulse             = 5.6[us]
Irr_mode            = Off
Tri_mode            = Off
Dante_presat        = FALSE
Initial_wait        = 1[s]
Recvr_gain          = 50
Relaxation_delay    = 5[s]
Repetition_time     = 6.4548992[s]
Temp_get            = 21.1[dC]
```

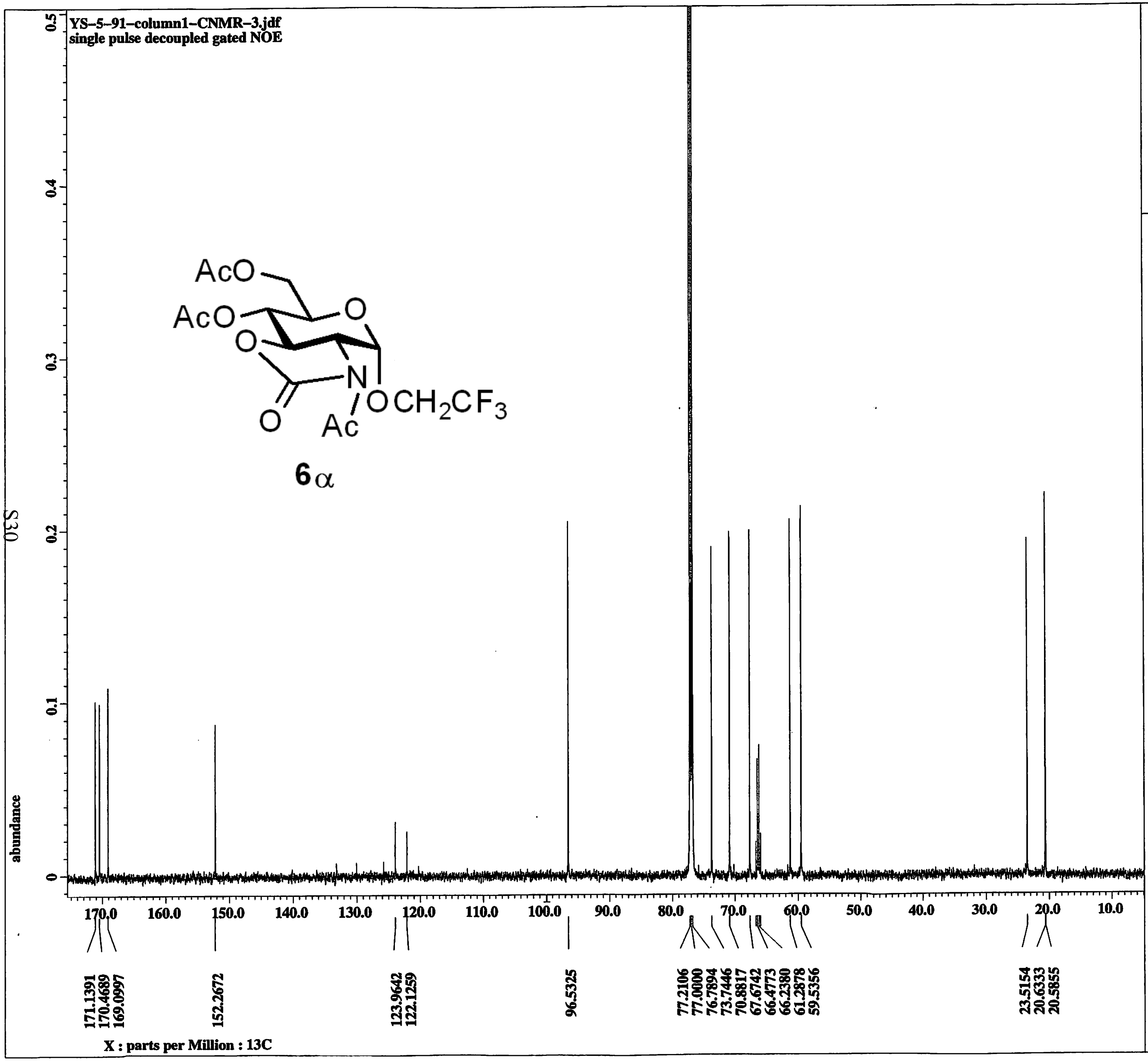


```
---- PROCESSING PARAMETERS ----
dc_balance : 0 : FALSE
sexp : 2.0[Hz] : 0.0[s]
trapezoid3 : 0[%] : 80[%] : 100[%]
zerofill : 1
fft : 1 : TRUE : TRUE
machinephase
ppm

Derived from: YS-5-91-column1-CNMR-1.jdf
```

```
Filename            = YS-5-91-column1-CNMR-
Author              = delta
Experiment          = single_pulse_dec
Sample_id           = YS-5-91-column1
Solvent             = CHLOROFORM-D
Creation_time       = 21-DEC-2011 00:57:42
Revision_time       = 21-DEC-2011 11:03:01
Current_time        = 21-DEC-2011 11:03:11

Content             = single pulse decouple
Data_format         = 1D COMPLEX
Dim_size            = 26214
Dim_title           = 13C
Dim_units           = [ppm]
Dimensions          = X
Site                = ECA600
Spectrometer        = JNM-ECA600

Field_strength      = 14.09636928[T] (600[M
X_acq_duration      = 0.69206016[s]
X_domain            = 13C
X_freq              = 150.91343039[MHz]
X_offset            = 100[ppm]
X_points            = 32768
X_prescans          = 4
X_resolution        = 1.44496109[Hz]
X_sweep             = 47.34848485[kHz]
Irr_domain          = 1H
Irr_freq            = 600.1723046[MHz]
Irr_offset          = 5[ppm]
Clipped             = TRUE
Mod_return          = 1
Scans               = 5000
Total_scans         = 5000

X_90_width          = 10.5[us]
X_acq_time          = 0.69206016[s]
X_angle             = 30[deg]
X_atn               = 8[dB]
X_pulse             = 3.5[us]
Irr_atn_dec         = 21.494[dB]
Irr_atn_noe         = 21.494[dB]
Irr_noise           = WALTZ
Decoupling          = TRUE
Initial_wait        = 1[s]
Noe                 = TRUE
Noe_time            = 2[s]
Recvr_gain          = 60
Relaxation_delay    = 2[s]
Repetition_time     = 2.69206016[s]
Temp_get            = 21.9[dC]
```

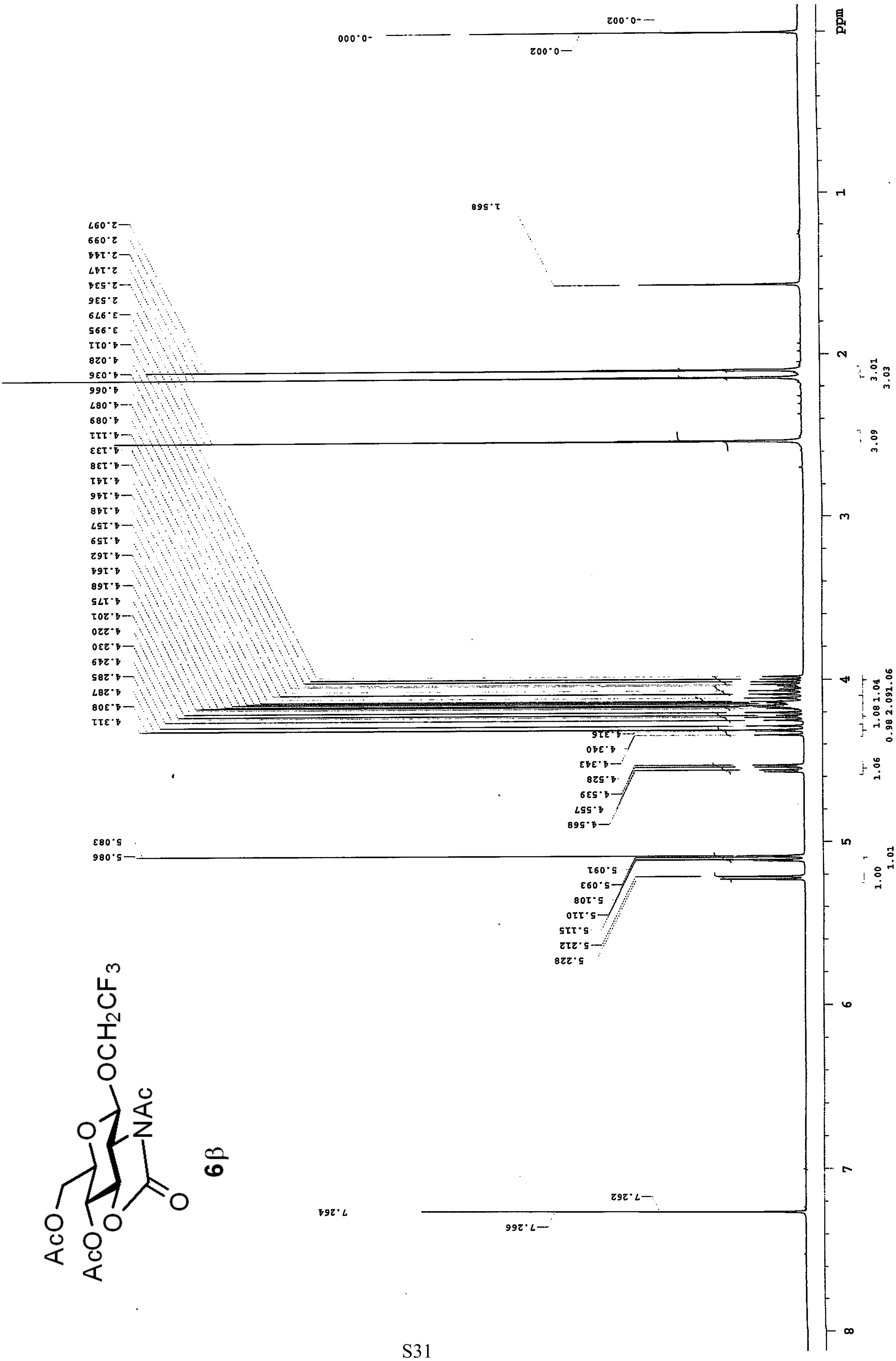

6β

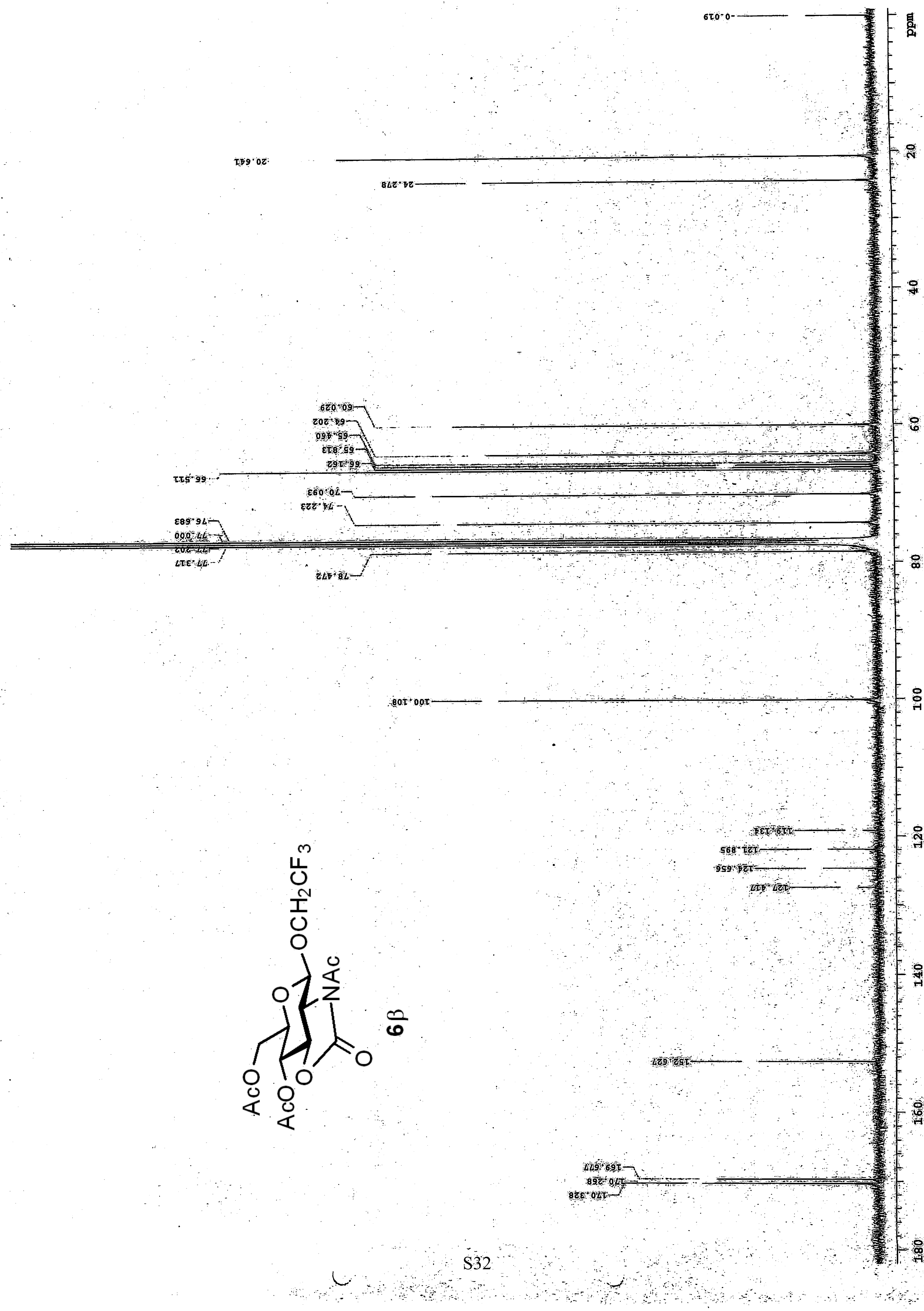

0.019
20.641
24.278
60.029
64.202
65.460
65.813
66.162
66.511
70.093
74.223
76.683
77.000
77.317
78.472
100.108
119.134
121.895
124.656
127.417
152.627
169.677
170.268
170.328
ppm
20
40
60
80
100
120
140
160
180
AcO
AcO
NAc
OCH2CF3

6β

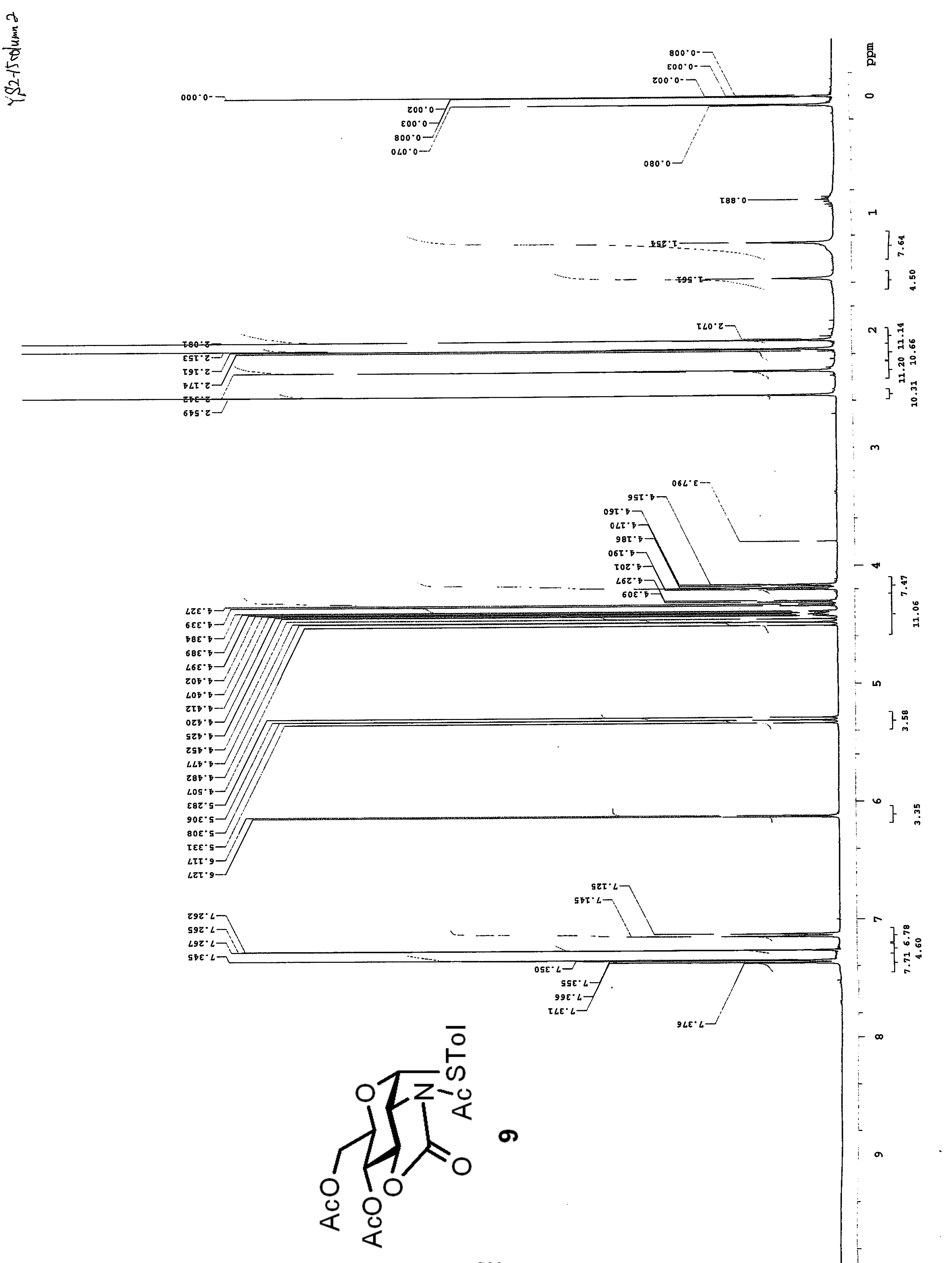
ppm
AcO
AcO
N
Ac
STol
9

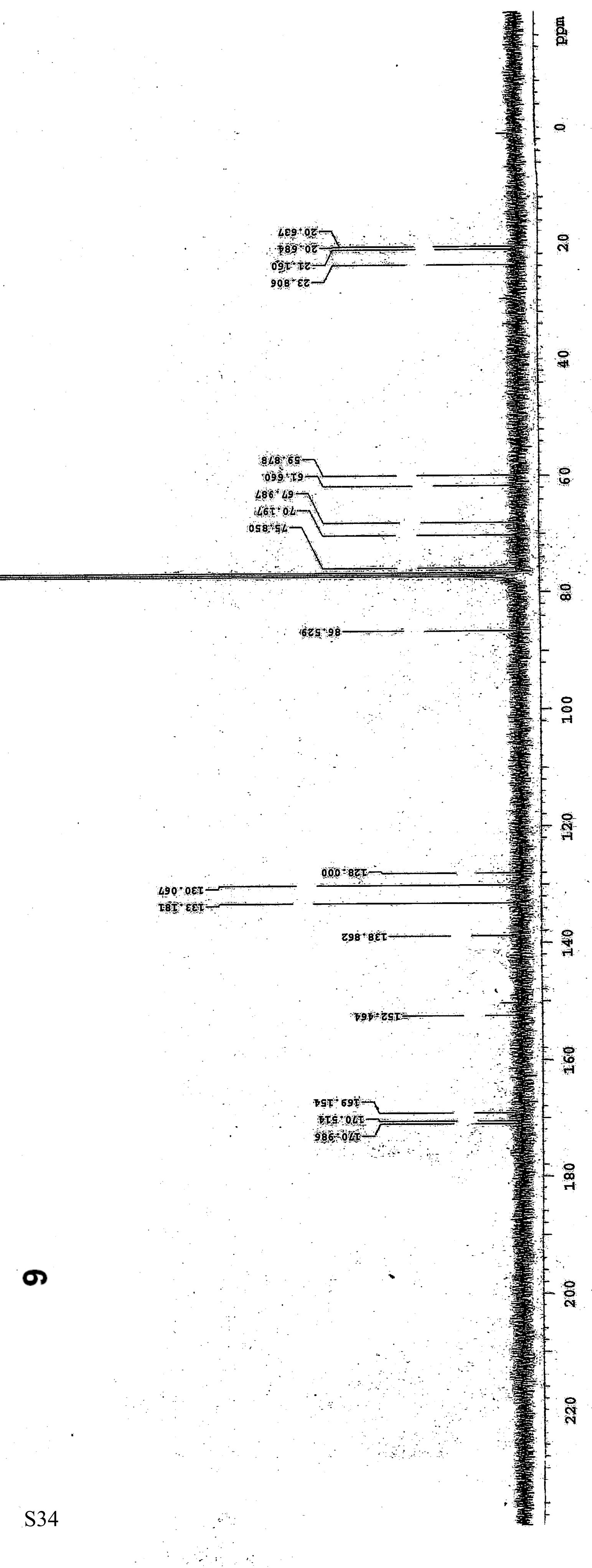

20.637
20.684
21.160
23.806
59.878
61.660
67.987
70.197
75.850
76.683
77.000
77.317
86.529
128.000
130.067
133.181
138.862
152.464
169.154
170.514
170.986
ppm

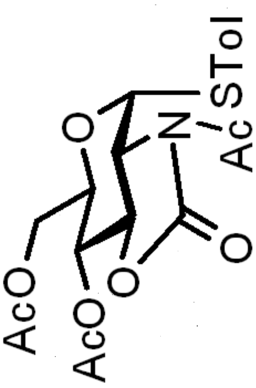

AcO
AcO
O
N
Ac
STol
O
O

9

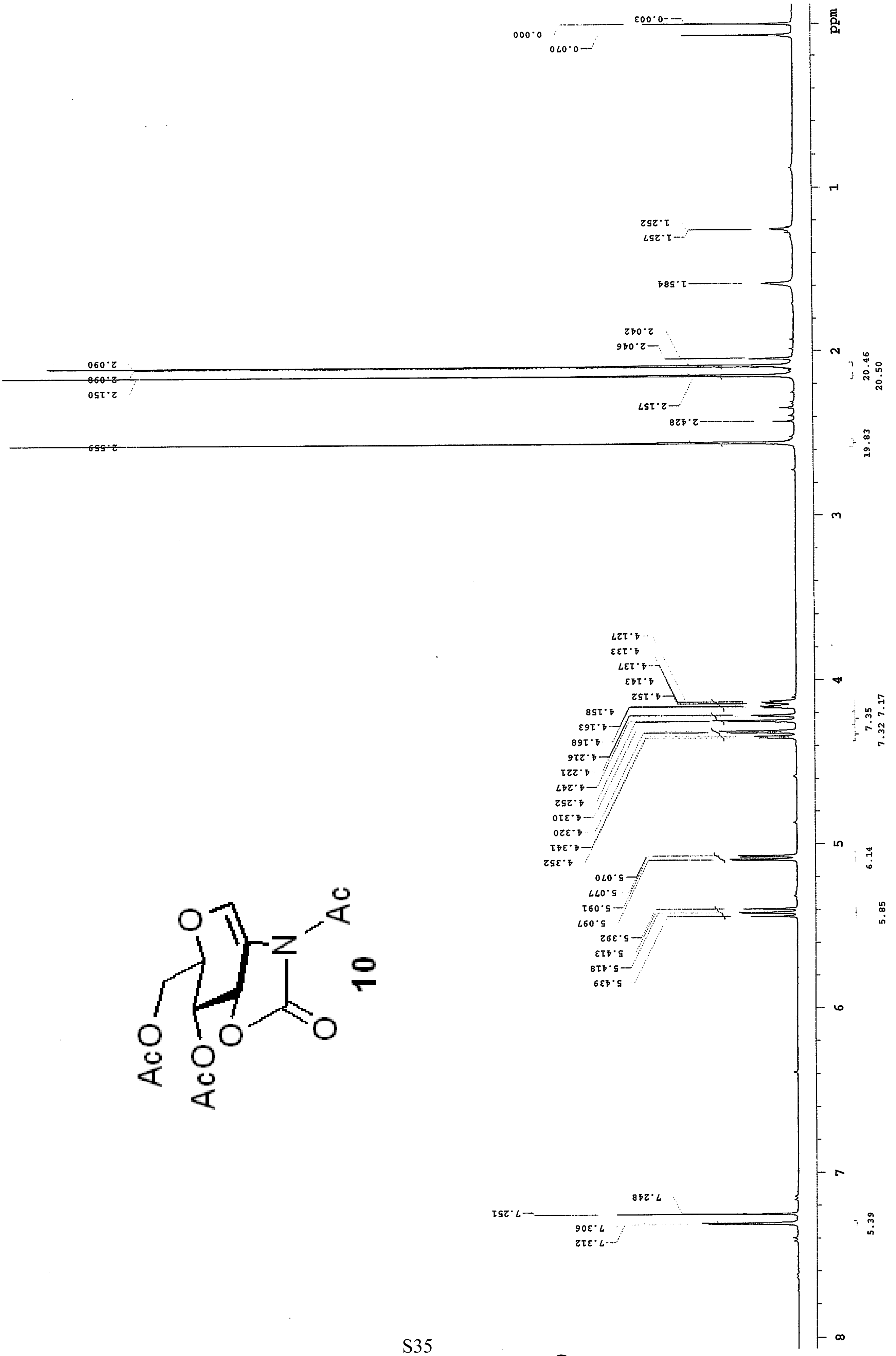

10

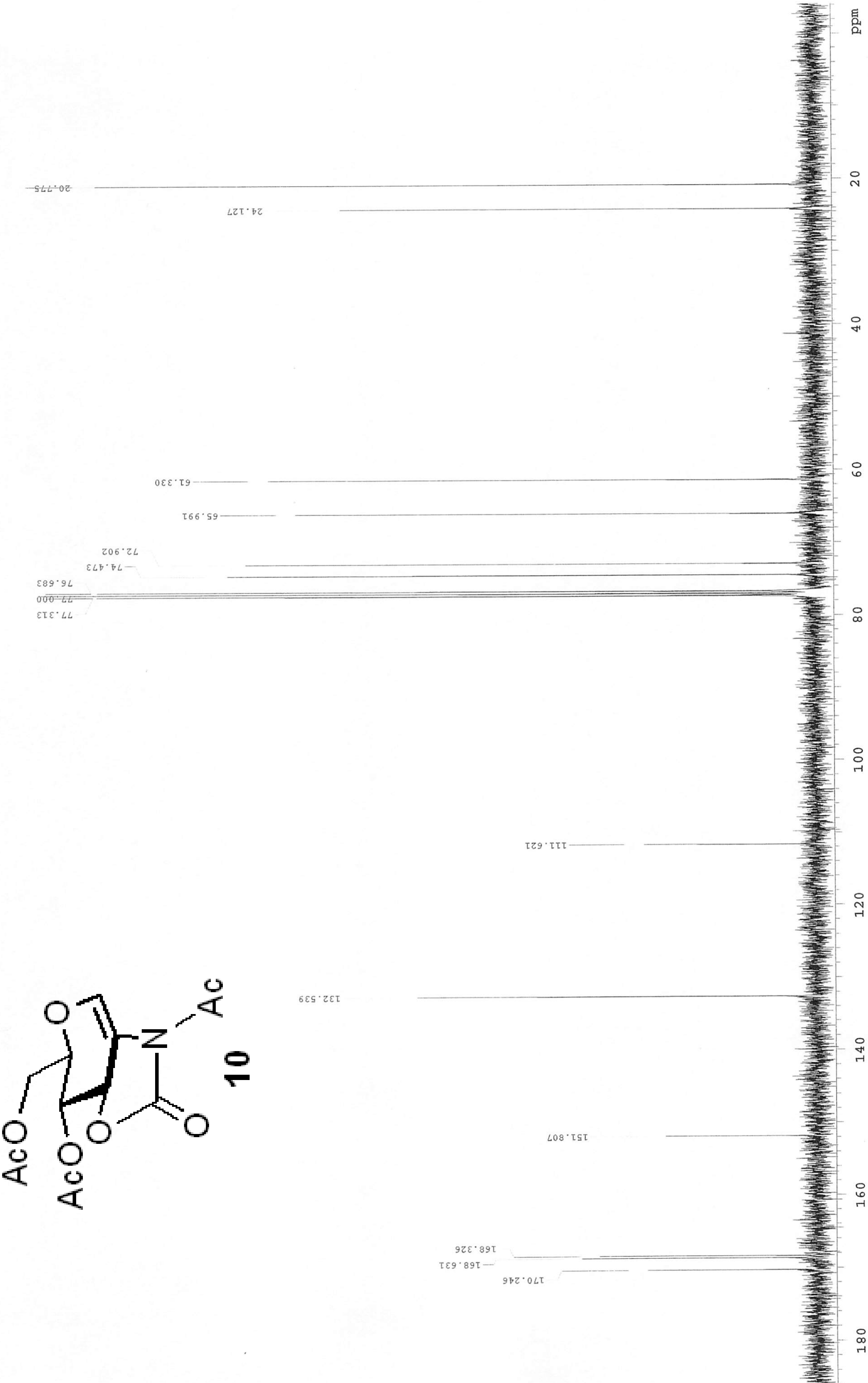
20.775
24.127
61.330
65.991
72.902
74.473
76.683
77.000
77.313
111.621
132.539
151.807
168.326
168.631
170.246
ppm
20
40
60
80
100
120
140
160
180
AcO
AcO
O
O
N
Ac
O
10
